# Performance and Health in Combined Events: A Scoping Review

**DOI:** 10.1111/sms.70190

**Published:** 2025-12-31

**Authors:** Pascal Edouard

**Affiliations:** ^1^ Laboratoire Interuniversitaire de Biologie de la Motricité Université Jean Monnet Saint‐Etienne, Lyon 1, Université Savoie Mont‐Blanc Saint‐Etienne France; ^2^ Department of Clinical and Exercise Physiology, Sports Medicine Unity University Hospital of Saint‐Etienne, Faculty of Medicine Saint‐Etienne France; ^3^ European Athletics Medical & Anti‐Doping Commission European Athletics Association (EAA) Lausanne Switzerland

**Keywords:** decathlon, epidemiology, health, heptathlon, illnesses, injuries, sports performance, sports science, track and field

## Abstract

Combined events are an Athletics discipline with specific and particular challenges for performance and health, supporting the interest of focused research on this discipline, despite concerning a small proportion of athletes. The study aim was to summarize and map the available scientific literature on performance and health of combined events to establish the current level of understanding and identify knowledge gaps that require further investigation. A scoping review was conducted searching peer‐reviewed articles dealing with performance and/or health in combined events (i.e., pentathlon, heptathlon or decathlon) on the MEDLINE (via PubMed), EMBASE (via Ovid), Web of Science, and Google Scholar databases, from inception to October 13, 2025. In total, 111 articles were included, with 95.5% as primary research, 95.5% using quantitative approach, 22.5% with a level of evidence 1b and 48.6% 2b, and 56.8% with study aim(s) focused on combined events understanding and/or analyzing. 64.0% articles dealt with performance and 59.5% with health, including 23.4% dealing with both. Regarding performance, the majority of articles dealt with performance analysis/tactics/data management (56.3%), followed by physiology (21.1%), and nutrition (11.3%). Regarding health, the majority of articles dealt with injuries (62.1%), followed by physiology (22.7%), illnesses (18.2%), and nutrition (12.1%). These findings (i) can help to suggest some clinical implications for performance enhancement and health protection, and (ii) highlighted the need for continuing research on performance and/or health in combined events, preferably with prospective design, large athletes' sample sizes, focused on underrepresented populations (e.g., women, adolescents, Masters athletes), over one or more Athletics season.

## 1 Introduction

Combined events are a particular discipline of Athletics, as athletes should compete in different running, jumping, and throwing events to complete their performance [1, 2]. Combined events are practiced worldwide. Over one or two consecutive days, athletes have to compete in 5–10 events, in heptathlon (outdoor) and pentathlon (indoor) for women and in decathlon (outdoor) or heptathlon (indoor) for men (Table 1) [2, 3]. In each event, athletes have to earn points by their performance and the sum of these points corresponds to the overall performance and determines the athletes' ranking [1, 2]. Thus, completing—and collecting points in—all events represents the first goal to reach an optimal performance and ranking [1, 2]. The challenge of performance in combined events is therefore complex, as athletes have to perform well in each single event as well as over one or two consecutive days. This warrants a combination of physical and physiological (e.g., speed, strength, flexibility, coordination and endurance), technical, and psychological capabilities [1, 2]. Zarnowski [1] therefore described the decathlon as “a menu of athletic events, testing an individual's speed, endurance, strength, skill, and personality.” Combined events are not merely a collection of individual events and they have specific demands which exceed the individual demands of each event. Information on performance‐related factors and training approaches from each specific event could be relevant to help combined events athletes in their performance preparation. Nevertheless, information and data specifically for combined events are of major interest given the specificities of combined events [4]. Thus, gathering all available scientific evidence about performance in combined events would allow determine the current knowledge, which in turn can help athletes, coaches, and stakeholders around them. This can also help to determine the knowledge gaps that require further investigation.

**TABLE 1:** Details of the Athletics discipline of combined events according to the sex (women and men) and the type of championship (outdoor and indoor), for competitive adult athletes, presented in the official order for competitions.

Sex	Type of championship		Number of events	First day					Second day				
Women	Outdoor	Heptathlon	7	100 m hurdles	High jump	Shot put	200 m sprint		Long jump	Javelin throw	800 m run		
	Indoor	Pentathlon	5	60 m hurdles	High jump	Shot put	Long jump	800 m run	—				
Men	Outdoor	Decathlon	10	100 m sprint	Long jump	Shot put	High jump	400 m sprint	110 m hurdles	Discus throw	Pole Vault	Javelin throw	1500 m run
	Indoor	Heptathlon	7	60 m sprint	Long jump	Shot put	High jump		60 m hurdles	Pole Vault	1000 m run		

Among the factors contributing to sporting performance, health can be considered as the cornerstone. For instance in alpine skiing, performance and health have been reported as pillars of achieving athletes' best condition to perform [5]. In addition, despite training for combined events could lead to health benefits, as any physical activity [6], it is also associated with negative consequences (e.g., injuries [7], illnesses [8]). These negative consequences can affect the athletes beyond their sport participation, on their daily life and after their career. Thus, potential health‐related problems associated with combined events have their own consequences and implications in the short, middle and long term, in addition to consequences on sport participation and performance. It is therefore of interest to better understand the trade‐off between health benefits and risks of combined events to protect and promote participation. Thus, both indirectly for performance and directly for athletes' health, there is an interest to collect all available scientific evidence on health in combined events. Among health aspects, injury represents an important one for the combined events participation and performance, and for long‐term health [3, 9]. During international Athletics championships, combined events was the discipline with the highest number of injuries per athlete [7]. During these championships, being injured during a combined events competition was associated with performance failure [3]. This supports the need to reduce injury risk in combined events for both performance and health perspectives, which in turn supports the need to gather evidence on injuries and their prevention.

Therefore, combined events are an Athletics discipline with specific and particular challenges for performance and health, supporting the interest of focused research on this discipline, despite concerning a small proportion of Athletics athletes [1]. In this context, the aim of this study was to summarize and map, through a scoping review, the available scientific literature on performance and health of combined events to establish the current level of understanding and identify knowledge gaps that require further investigation.

## 2 Methods

### 2.1 Protocol and Registration

A scoping review was conducted since this approach is superior to a systematic review for addressing an exploratory research question [10]. The reporting of this scoping review adheres to the Preferred Reporting Items for Systematic Reviews and Meta‐Analyses Statement extension for Scoping Reviews (PRISMA‐ScR) guidelines [11]. The protocol of this scoping review was a priori registered on Open Science Foundation (https://doi.org/10.17605/OSF.IO/S32MN).

### 2.2 Eligibility Criteria

Articles were included if they:

– dealt with the Athletics discipline of combined events (i.e., pentathlon, heptathlon or decathlon (Table 1));
– concerned or involved competitive human athletes competing in combined events;
– provided data or information about performance and/or health;
– when athletes from different Athletics disciplines and/or sports were included, included specific data or information on combined events;
– were published in English, French, German, Italian, Portuguese, and Spanish languages;
– were peer‐reviewed, including original research, systematic reviews, meta‐analyses, scoping reviews, guidelines, research protocols, editorials, opinion pieces, commentaries, letters to the editor, descriptive studies, anecdotal studies, case series and case reports;
– and only if their full texts were available.

Articles were excluded if they:

– were non‐peer‐reviewed articles (e.g., newspaper articles, theses, conference proceedings/abstracts, book chapters).

### 2.3 Information Sources and Search

A comprehensive search of the literature was undertaken on the MEDLINE (via PubMed), EMBASE (via Ovid), Web of Science, and Google Scholar databases, as recommended by Bramer et al. [12], from inception to October 13, 2025. The search strategy was initially developed for PubMed (Table 2), and subsequently adapted for each database. The keywords were inspired from Ma et al. [13] to explore performance, and from Murray et al. [14] to explore health. The complete search string of each database is provided in Table S1.

**TABLE 2:** Research equation developed and used for the MEDLINE (via PubMed) from inception to October 13, 2025.

#1	“combined events”[All Fields] OR “decathlon”[All Fields] OR “heptathlon”[All Fields] OR “pentathlon” [All Fields] OR “decathlete*”[All Fields] OR “heptathlete*”[All Fields] OR “pentathlete*”[All Fields]
#2	“performance” OR “athletic performance” OR “sport performance” OR “physical performance” OR “training effects” OR “performance improvement” OR “performance enhancement” OR “physical fitness” OR “strength” OR “flexibility” OR “agility” OR “speed” OR “power” OR “endurance” OR “coordination” OR “balance” OR “technical skills” OR “technique” OR “technical performance” OR “skill” OR “skill development” OR “skill performance”
#3	“health*” OR “injur*” OR “illness*” OR “fitness” OR “morbidit*” OR “mortalit*” OR “well‐being” OR “longevit*” OR “body composition*” OR “life expectanc*” OR “mental health” OR “wellness” OR “cardiovascular*”
#1 AND (#2 OR #3) No filters were used in the electronic searches.	

### 2.4 Selection of Sources of Evidence

All records selected from the electronic searches were included on Rayyan (https://new.rayyan.ai) [15] to check for redundancy, and duplicates were automatically detected and manually deleted. One independent reviewer (PE) screened records for eligibility, first based on the title and abstract, and then based on the full text also using Rayyan [15]. Additionally, the reference lists of included articles were manually screened to identify any additional relevant articles.

### 2.5 Data Charting Process and Data Items

One reviewer (PE) independently extracted data based on the following categories: (a) first author and year of publication; (b) type of research (*primary research* (i.e., original research) when the author(s) of the source generated the data, or *secondary research* when the author(s) of the source did not generate the data); (c) research approach (quantitative or qualitative); (d) study design approach (descriptive or analytic); (e) study design (prospective, retrospective or cross‐sectional); (f) level of evidence [16]; (g) aim or focus; (h) combined events explored (combined events in general, heptathlon (women outdoor), pentathlon (women indoor), decathlon (men outdoor), and/or heptathlon (men indoor)); (i) characteristics of the population (sample size, country, gender); (j) domain: performance and/or health; (k) key findings that relate to the scoping review research questions.

Articles in the domain of performance were then classified in: physiology, genetics, biomechanics, nutrition, motor control/skill acquisition, strength and conditioning/motor development/training, psychology, performance analysis/tactics/data management, and/or technology, inspired from the classifications proposed by Smith [17], Glazier [18] and Bangsbo et al. [19].

Articles in the domain of health were then classified according to the biopsychosocial model from Engel [20]. The articles classified in “biological” were sub‐classified in: physiology (corresponding to non‐pathological aspects related to health (e.g., cardiovascular physiology, energy expenditure, biomechanics)), nutrition, illnesses (corresponding to non‐musculoskeletal pathologies (e.g., cardiovascular, respiratory, metabolic diseases)), and/or injuries (corresponding to musculoskeletal pathologies). As injury prevention represents an important challenge in sports, articles concerning injuries were also sub‐classified according to the 6 stages of the TRIPP framework (i.e., stage 1: injury surveillance; stage 2: establish etiology and mechanisms of injury; stage 3: develop preventive measures; stage 4: “Ideal conditions”/scientific evaluation; stage 5: describe intervention context to inform implementation strategies; stage 6: evaluate effectiveness or preventive measures in implementation context) [21].

### 2.6 Critical Appraisal of Individual Sources of Evidence

To better summarize and map the available literature on combined events regarding performance and health, the Level of Evidence of each article was determined as per the guidelines of the Centre for Evidence‐Based Medicine Oxford Levels of Evidence [16]. The Level of Evidence was not extracted but evaluated.

### 2.7 Synthesis of Results

First, a descriptive analysis of the extracted data was performed using frequency and percentages for categorical variables, and means and standard deviations (SD) for continuous variables. Then, a thematic summary was performed providing a descriptive analysis on how the identified research relates to the research question and the main findings from these, organized by theme [14]. This allowed to (i) map the evidence and key concepts available on combined events and performance and/or health, (ii) summarize existing research findings, and (iii) identify research gaps in the existing literature [14].

## 3 Results

### 3.1 Articles Selection

**FIGURE 1:**
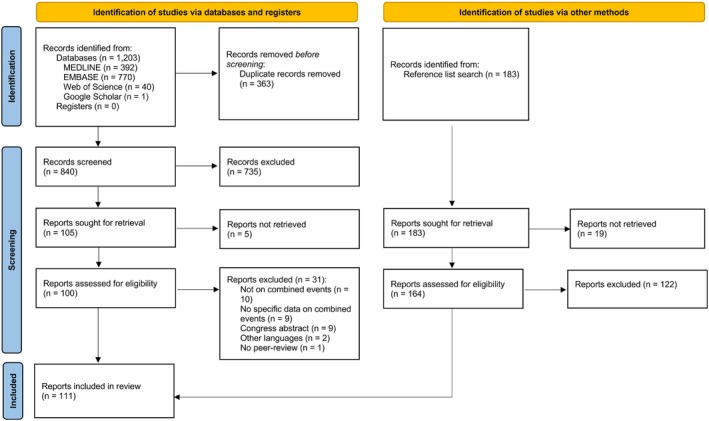
Preferred Reporting Items for Systematic Reviews and Meta‐Analysis (PRISMA) flow chart of the selection of the articles [10].

The initial search in the 4 databases yielded 1203 references, from which 363 duplicates were removed (Figure 1). A total of 840 references were screened based on title and abstract, from which 105 were selected for the full text screening, resulting in 69 included articles (Figure 1). The list of excluded articles due to unavailability of the full text (*n* = 5) and those excluded after full text screening (*n* = 31) is available in Table S2. Additionally, 42 articles were included through reference lists' screening of already included articles. In total, 111 articles were included (Figure 1).

### 3.2 Characteristics of the Included Articles

The main characteristics of the 111 included articles are presented in the Table S3. Almost all included articles were primary research (*n* = 106; 95.5%), including 100 (90.1%) original research and 6 (5.4%) case reports [22, 23, 24, 25, 26, 27], while 5 articles were secondary research (4.5%), all being narrative reviews [28, 29, 30, 31, 32]. The research approach was quantitative for 106 articles (95.5%), qualitative for 1 (0.9%) article [33], and not applicable for 4 articles (3.6%). The majority of included articles aimed at a descriptive approach (*n* = 68; 61.3%), followed by an analytic approach (*n* = 40; 36.0%). The study design was prospective for 37 (33.3%) articles, retrospective for 49 (44.1%), cross‐sectional for 20 (18.0%), and not applicable for the 5 narrative reviews (4.5%). The level of evidence was 1b for 25 (22.5%) articles, 2b for 54 (48.6%), 3 for 17 (15.3%), 4 for 10 (9.0%), and 5 for 5 (4.5%).

The oldest article dated back from 1953 [34], while most of the articles (38.7%; *n* = 43) were published in the last 10 years (Figure S1).

A total of 97 (87.4%) articles reported the number of athletes included, 9 (8.1%) articles did not report it, and there were 5 (4.5%) narrative reviews without included athletes. The number of athletes per article ranged from 1 to 3103, for a total of 17 181 included athletes. Among these 97 articles, 16 (14.4%) only included women, 54 (48.6%) only included men, and 29 (26.1%) included both women and men, while 9 (8.1%) of the latter did not specify the number of athletes by sex. Of the 88 articles reporting sex‐specific number of athletes, 36 (32.4%) included women for a total of 2518 included women athletes, and 72 (64.9%) included men with a total of 13 079 included men athletes.

Geographically, most of the articles were performed on international cohorts including athletes from all continents and countries (*n* = 49; 44.1%), while in other articles, athletes originated mostly from the USA (*n* = 13; 11.7%) and France (*n* = 11; 9.9%) (Figure S2).

### 3.3 Domains of Included Articles

For 56.8% (*n* = 63) of included articles, the study aim(s) focused on combined events understanding and/or analyzing, while for other articles (43.2%) the study aimed at many Athletics disciplines and/or sports including combined events, or used combined events as an experimental model.

**FIGURE 2:**
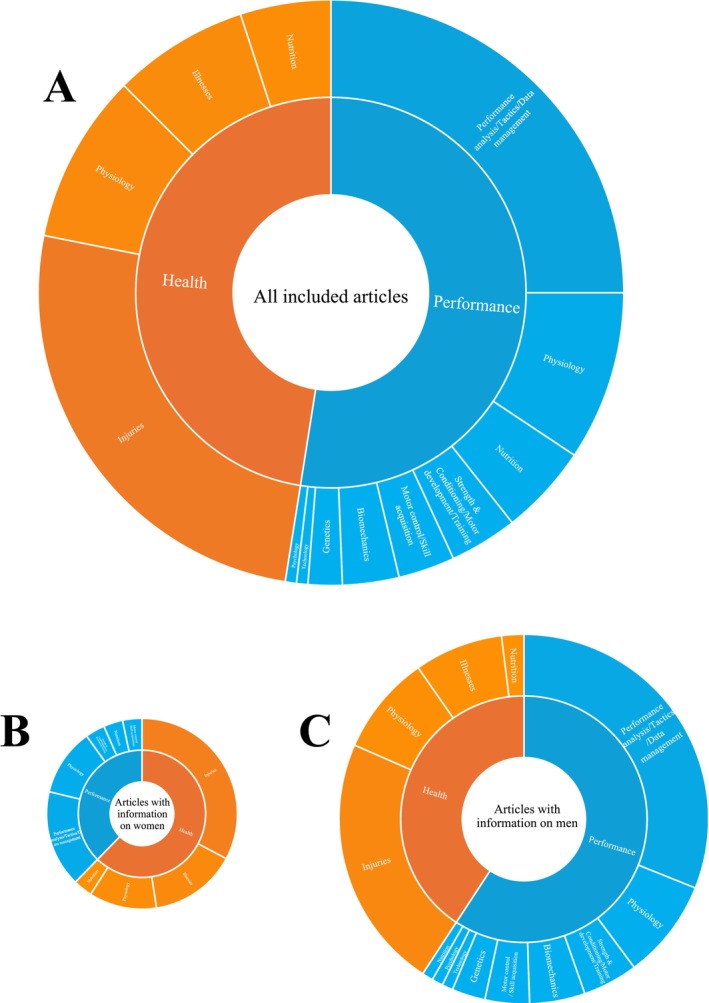
Distribution of the domains (i.e., performance and health) and sub‐domains for the 111 included articles (A), and for articles included information on women (*n* = 36) (B) and on men (*n* = 72) (C). The size of the figures is proportional to the number of articles.

A total of 26 (23.4%) articles dealt with both performance and health, 45 (40.5%) only with performance, and 40 (36.0%) only with health, corresponding to a total of 71 (64.0%) articles dealing with performance and 66 (59.5%) with health (Figure 2 and Table 3).

**TABLE 3:** Classification of the 111 included studies according to the domains.

Domains and sub‐domains	Number of articles (%)	References	Level of evidence (range)
Performance	71 (64.0)		1b to 4
Physiology	15 (22.1)	[35, 36, 37, 38, 39, 40, 41, 42, 43, 44, 45, 46, 47, 48, 49]	1b to 4
Genetics	3 (4.2)	[50, 51, 52]	2b to 3
Biomechanics	5 (7.0)	[53, 54, 55, 56, 57]	2b to 4
Nutrition	8 (11.3)	[28, 29, 32, 40, 41, 43, 58, 59]	2b to 5
Motor control/Skill acquisition	4 (5.6)	[57, 60, 61, 62]	2b to 3
Strength and conditioning/Motor development/Training	6 (8.5)	[55, 56, 63, 64, 65, 66]	2b to 4
Psychology	1 (1.4)	[33]	1b
Performance analysis/Tactics/Data management	40 (56.3)	[3, 34, 52, 60, 62, 64, 67, 68, 69, 70, 71, 72, 73, 74, 75, 76, 77, 78, 79, 80, 81, 82, 83, 84, 85, 86, 87, 88, 89, 90, 91, 92, 93, 94, 95, 96, 97, 98, 99, 100]	1b to 3
Technology	1 (1.4)	[101]	1b
Health	66 (59.5)		
Biological aspect	66 (100.0)		
Physiology	15 (22.7)	[35, 36, 37, 38, 39, 40, 41, 42, 43, 44, 45, 46, 47, 48, 49]	1b to 4
Nutrition	8 (12.1)	[28, 29, 32, 40, 41, 43, 58, 59]	2b to 5
Illnesses	12 (18.2)	[8, 27, 58, 63, 65, 102, 103, 104, 105, 106, 107, 108]	1b to 4
Injuries	41 (62.1)	[3, 7, 9, 22, 23, 24, 25, 26, 27, 30, 31, 62, 65, 66, 67, 97, 107, 109, 110, 111, 112, 113, 114, 115, 116, 117, 118, 119, 120, 121, 122, 123, 124, 125, 126, 127, 128]	1b to 5
Stage 1: injury surveillance	36 (87.8)	[3, 7, 9, 25, 30, 31, 62, 65, 66, 67, 97, 107, 109, 110, 111, 112, 113, 114, 115, 116, 117, 118, 119, 120, 121, 122, 123, 124, 125, 126, 127, 128]	1b to 5
Stage 2: establish etiology and mechanisms of injury	9 (22.0)	[65, 106, 107, 110, 113, 115, 119, 122, 128]	1b to 2b
Stage 3 to 6	0 (0.0)		
Psychological aspect	1 (1.5%)	[67]	2b
Environmental aspect	2 (3.0%)	[63, 102]	1b to 2b

Among the 71 articles dealing with performance, the majority dealt with performance analysis/tactics/data management (*n* = 40; 56.3%), followed by physiology (*n* = 15; 21.1%), and nutrition (*n* = 8; 11.3%) (Figure 2 and Table 3).

Among the 66 articles dealing with health, all dealt with biological aspects (*n* = 66, 100.0%), while 1 (1.5%) also dealt with psychological [67] and 2 (3.0%) also with environmental aspects [63, 102] (Figure 2 and Table 3). Then, among the 66 articles dealing with biological aspects, the majority of articles dealt with injuries (*n* = 41; 62.1%), followed by physiology (*n* = 15; 22.7%), illnesses (*n* = 12; 18.2%), and nutrition (*n* = 8; 12.1%) (Figure 2 and Table 3). For the 40 articles dealing with injuries, 36 (87.8%) articles dealt with the stage 1: injury surveillance, and 9 (22.0%) with stage 2: establish etiology and mechanisms of injury, and no articles dealt with the stages 3 to 6 of the TRIPP framework.

### 3.4 Thematic Analysis

#### 3.4.1 Performance

##### 3.4.1.1 Physiology

The articles dealing with physiology reported data on body composition (e.g., body fat mass, body fat‐free) (*n* = 9) [35, 36, 37, 38, 39, 40, 41, 42, 43, 44], maximal oxygen consumption (VO_2_max) (*n* = 6) [35, 36, 37, 45, 46, 47], strength (*n* = 3) [35, 36, 45], respiratory function (*n* = 2) [36, 37], cardiac function (*n* = 1) [46], muscle anthropometry (*n* = 1) [39], muscle histochemistry (*n* = 1) [45], bone mineral density and bone metabolism (*n* = 1) [42], blood lactate during a decathlon (*n* = 1) [37], heart rate during a decathlon (*n* = 1) [48], and blood and urine markers of oxidative stress during a heptathlon (*n* = 1) [49]. The mean values of fat mass percentage were 11%–15% in heptathletes (women) [35, 40, 41], and 8%–14% in decathletes [36, 37, 38, 39, 42, 43, 44]. The mean values of maximal oxygen consumption were 40–50 mL/kg/min for heptathletes (women) [35, 45, 47], and 52–58 mL/kg/min for decathletes [36, 37, 46]. Maïmoun et al. [42] reported higher bone mineral density and higher bone turnover in 13 national‐ to international‐level decathletes compared to 13 healthy control participants, suggesting that training for decathlon seemed particularly osteogenic.

##### 3.4.1.2 Genetics

The genetic analysis of national‐ and international‐level decathletes reported higher prevalence of polymorphisms associated with improved speed performance [50], strength performance [51, 52], power performance [52], and lactate transport [51], compared to other Athletics athletes or controls.

##### 3.4.1.3 Biomechanics

Kunz and Kaufmann reported differences between national‐level decathletes and event‐specialist world class athletes in the kinematics of a 100‐m race [53] and javelin throw [54], that could explain lower performance in decathletes. In six national‐ to international‐level decathletes, Edouard et al. [55] observed a significant correlation between the velocity in squat jump measured at the start of a decathlon and the final performance of the respective decathlon. Bogdanis et al. [56] reported the kinematic of long‐jump take‐off in eight national‐level decathletes. Horst et al. [57] were able to identify the kinematic patterns of the three decathlon throwing events independently of the athletes, in seven national‐team level decathletes.

##### 3.4.1.4 Nutrition

In 19 national‐ to international‐level heptathletes, Houtkooper et al. [40] reported a low body fat and high fat free mass relative to their height, and high levels of bone mineral density compared to standard bone mineral density values for women of their age and ethnicity. In 19 national‐ to international‐level heptathletes, Mullins et al. [41] reported that the average nutrient intakes exceeded the dietary reference intakes (> 67%), suggesting that they had adequate nutrient intakes to promote glycogen availability, utilization, recovery and muscle tissue growth and repair. Tscholl et al. [58] reported that the frequencies of use of nutritional supplements were higher in combined events than in other Athletics disciplines. In Masters combined events athletes (*n* = 3), Leonhardt et al. [59] reported that, while they were competing at a world championship, for many they were not consuming the recommended levels of overall energy, carbohydrates, protein, and some micronutrients. In 10 beginner‐ to national‐level decathletes, Yoshitake et al. [43] explored the blood glucose levels and the timing of intakes during a decathlon, and reported both low and high blood glucose levels during a decathlon, with inter‐athlete variability, supporting the hypothesis that fluctuations in blood glucose levels stemmed from repeated cycles of food intakes and exercises. Three narrative reviews [28, 29, 32] provided recommendations for nutrition and hydration in combined events training and competition mainly based on the general knowledge about nutrition and hydration according to the type of exercises and sports demands. Information from narrative reviews was complementary to that from included articles about nutrition.

##### 3.4.1.5 Motor Control/Skill Acquisition

Careau et al. [60] reported that performance in all throwing events increased with aging, while performance in running/jumping events (i.e., 100 m, hurdles, 400 m, 200 m, long jump, high jump, 1500 m and 800 m) decreased with aging, by analyzing data of 3266 heptathlon and 2625 decathlon performances. Reuter et al. [61] reported no correlation between the Star Excursion Balance Test (SEBT) and any of the Single‐Leg‐Hop Tests (SLHT) in seven national‐team level decathletes. Horst et al. [57] reported that certain individual movement characteristics can be identified in the kinematic patterns of both shot put and discus throwing. Chapon et al. [62] descriptively reported the evolution of combined events performance with aging in 3 national‐level decathletes and one national‐level heptathlete.

##### 3.4.1.6 Strength and Conditioning/Motor Development/Training

Edouard et al. [55] reported no differences in lower extremity power output and their force and velocity components, measured by squat‐jump and cycling sprint, between all four testing measurements (i.e., before and after each of the 2 days of a decathlon), suggesting that decathlon cannot be compared to repeated‐sprint exercises, but rather to an intermittent‐maximal exercise. Bogdanis et al. [56] reported the interest of plyometric conditioning exercise executed 3 min before each attempt in order to increase long‐jump performance. In relation to heat, Périard et al. [63] reported that 16.7% of international‐level combined events athletes (*n* = 12) reported following a heat acclimatization regimen, with a mean length of 30 ± 0 days before the championship. Using the performance records of 446 top‐athletes from 20 to 74 years old, Panday et al. [64] reported that aging induces a gradual decrease in performance in 100, 400 and 1500 m, and suggested that motor ability structure suddenly changes around the age of 50. Edouard et al. [65] reported that international‐level heptathletes and decathletes (*n* = 13) trained an average 11.4 ± 2.6 and 14.8 ± 10.3 h per week, respectively, during the month before a European Athletics championship. These athletes also reported doing regularly (between > 1/month to > 3/week) hamstring strengthening and stretching, core stability and maximal sprinting, and they were those, among the 357 participating athletes, who did these exercises most frequently [65]. Edouard et al. [66] reported the proportion of athletes who dropped out of Athletics participation, where the proportion among combined events athletes (*n* = 51) was the second highest (27.5%) after the throwers (28.9%).

##### 3.4.1.7 Psychology

Dale [33] reported, in a qualitative analysis through interviews of seven international‐level decathletes who scored ≥ 8000 points during decathlon competitions, that distractions (i.e., lack of confidence, fatigue, bad event, pain, fear, weather, other competitors, the 1500 m) and coping strategies employed to deal with these distractions (i.e., imaging/visualization, being aware of cues, competing only against self, confidence in one's training, consistency, camaraderie) were the two major themes emerging from their “most memorable performance.”

##### 3.4.1.8 Performance Analysis/Tactics/Data Management

Several articles analyzed the determinants of performance in combined events through the analysis of performance data (e.g., total and by event performance) using factor analysis [34, 68, 69, 70, 71, 72, 73, 74, 75], clustering [76, 77, 78, 79, 80, 81, 82, 83], principal component analysis [64, 84, 85, 86, 87, 88], regression [3, 60, 69, 75, 89, 90, 91, 92], correlation [52, 69, 70, 72, 74, 80, 84, 90, 91, 93, 94], and/or descriptive [62, 67, 95, 96, 97] approaches. Seven used data from women heptathlon [3, 62, 76, 79, 86, 91, 97], one from women pentathlon [98], and 33 from decathlon [3, 34, 52, 60, 62, 64, 67, 68, 69, 70, 71, 72, 73, 75, 77, 78, 80, 81, 82, 84, 85, 87, 88, 89, 90, 92, 93, 94, 95, 96, 97, 99, 100] (note that three articles used both heptathlon and decathlon data [3, 62, 97]). In general, these articles highlighted some key events or key combinations of events for the success of a heptathlon, pentathlon or decathlon. These key events or key combinations of events slightly differed according to articles, sample of data, and/or analyses performed. For example, Ward et al. [84] reported that “the ten events in the Olympic decathlon cannot be reduced to a smaller set describing similar athletic versatility” and Kenny et al. [94] suggested that “to compete successfully at this level (i.e., Olympic level), a uniform, relatively high performance in all individual disciplines is required.” In agreement, Careau et al. [60] reported that “when an athlete performs better than usual in an event of a given competition, he/she tends to perform better than usual in all other events of the same competition.” In general, analyses showed that speed, explosive strength, technique and endurance represented the main factors of performance in combined events [34, 68, 73, 80, 81, 85, 89, 96]. Some articles reported the changes in combined events performance and in the distribution in points per events for performance with aging [60, 64, 88, 90, 92]. Three articles analyzed human performance trade‐offs through the analysis of performance in decathlon [60, 70, 93]. Van Damme et al. [93] concluded that “in an environment in which the selection criterion is combined high performance across multiple tasks, increased performance in one function may impede performance in others.” Careau et al. [60] reported that their results “verify that human performance is limited by fundamental genetic, environmental and aging constraints that preclude the simultaneous improvement of performance in multiple dimensions.”

##### 3.4.1.9 Technology

Purdy and White [101] published in 1976 the feasibility of using a portable minicomputer at the decathlon site to help eliminate errors in the administration of the event. Technological advances in combined events are not researched since then. Nowadays, using digital point systems in competition is a standard.

#### 3.4.2 Health

##### 3.4.2.1 Biological Aspects

###### 3.4.2.1.1 Physiology

The sixteen articles classified in the health sub‐domain of physiology were the same as those classified in the performance sub‐domain of physiology [35, 36, 37, 38, 39, 40, 41, 42, 43, 45, 46, 47, 48, 49, 61, 63].

###### 3.4.2.1.2 Nutrition

The eight articles classified in the health sub‐domain of nutrition were the same as those classified in the performance sub‐domain of nutrition [28, 29, 32, 40, 41, 43, 58, 59].

###### 3.4.2.1.3 Illnesses

Several articles reported the epidemiology of illnesses in combined events during international Athletics championships [8, 63, 65, 102, 103, 104, 105, 106, 107]. A combined analysis of illnesses during 11 international Athletics championships reported 33.5 illnesses per 1000 registered athletes in combined events, with higher rates (i) in women than men (49.1 vs. 19.7 illnesses per 1000 registered athletes), and (ii) during outdoor than indoor championships (41.2 vs. 14.5 illnesses per 1000 registered athletes) [8]. During the month before the international Athletics championships, a combined analysis of 7 international Athletics championships reported that, for combined events, 23.8% of women and 21.7% of men reported having experienced a pre‐participation illness complaint [107]. The characteristics of illnesses were not available specifically for combined events [8, 107]. Tscholl et al. [58] reported that the frequencies of use of medications (especially non‐steroidal anti‐inflammatory drugs and contraceptives) and nutritional supplements were higher in combined events than in other Athletics disciplines. Aguilar‐Navarro et al. [108] reported adverse analytical findings per drug class using data from the WADA‐accredited laboratories; in combined events there was 0.7% of adverse findings with higher numbers of hormone and metabolic modulators, anabolic agents and stimulants. In a case report of a heptathlete, Allen et al. [27] highlighted the role of sleep in the management of a medial tibial stress syndrome. None of the 12 articles [8, 27, 58, 63, 65, 102, 103, 104, 105, 106, 107, 108] dealing on illnesses in combined events had a study aim exclusively focused on combined events understanding and/or analyzing.

###### 3.4.2.1.4 Injuries

Data on injuries in combined events were reported in the context of (i) international Athletics championships [3, 7, 65, 107, 109, 110, 111, 112, 113, 114, 115, 116], with two articles focused only on combined events [3, 116], (ii) other championships/competitions [67, 97, 117, 118, 119], with two articles focused only on combined events [67, 97], (iii) one or more Athletics season with prospective data collection [62, 120, 121, 122], or (iv) a part, one or more Athletics season with retrospective data collection [9, 66, 123, 124, 125, 126, 127, 128]. Among the 41 articles dealing with injuries in combined events, the study was exclusively focused on combined events understanding and/or analyzing for only 9 (22.0%) [3, 9, 31, 67, 97, 116, 122, 123, 125]. During 14 international Athletics championships, a combined analysis of injuries in combined events reported 235 and 212 injuries per 1000 registered athletes in men and women, respectively, with higher rates during outdoor than indoor championships [7], and no differences between men and women [7, 113]. Combined events was the discipline with the highest number of injuries per 1000 registered athletes compared to all other Athletics disciplines [7]. Some events led to higher injury risk (i.e., pole vault and high jump for decathlon, and long jump for heptathlon) [116]. Regarding injury characteristics, there were no significant differences between men and women [7]. In men, most injuries were located at the thigh (19.5%), ankle (15.6%) or knee (14.3%), they affected muscles (29.9%), skin (22.1%) or tendons (18.2%), they were caused by trauma (42.9%) or overuse (36.4%) [7]. In women, most injuries were located at the thigh (21.5%), knee (16.9%) or trunk (13.8%), they affected muscles (38.5%), ligaments (27.7%) or tendons (12.3%), they were caused by trauma (40.0%) or overuse (40.0%) [7]. During the month before the international Athletics championships, a combined analysis of 7 international Athletics championships reported that, for combined events, 57% of women and 35% of men reported having experienced a pre‐participation injury complaint [107]. Two articles reported injury rates and characteristics in youth and junior combined events championships, highlighting high injury rates (i.e., similar or higher than in adults) [97, 111]. One article analyzed injuries during a championship of Masters (i.e., older than 35 years) and reported 4 injuries per 1000 registered athletes, where combined events were the discipline with the lowest injury rate, without any characteristics available [117]. During one or more Athletics season, articles reported that there were about 50% to 65% of injured athletes [121, 124], and about 2.7 to 3.3 injuries per athlete per season [62, 126]. Outside of the context of championships/competitions, injury characteristics were described only in retrospective studies, ranging from a part of one to many Athletics seasons [9, 123, 125, 126, 127]. Two articles analyzed potential relationships between performance and injuries [3, 62], with injuries being associated with lower performance [3]. There were two narrative reviews: one on injury prevention in Athletics with specific focus on injury epidemiology and risk factors in combined events [30], and one specifically on injuries in combined events [31]. There were also 6 case reports on specific injuries occurred in combined events athletes [22, 23, 24, 25, 26, 27], but the specificities of combined events was neither highlighted nor discussed. Among the 9 articles reporting information on the stage 2 of the TRIPP framework [65, 106, 107, 110, 113, 115, 119, 122, 128], only two reported information specifically for combined events: there were no sex‐related differences in injury risk during international Athletics championships [113], and muscle and hamstring injury proportion and rate was associated to the required velocity of the event [115].

##### 3.4.2.2 Psychological Aspects

The only article on the domain of health classified as dealing with psychological aspects was a pilot study, on 3 decathlons, including 50 athletes, aiming at determining the cause of dropouts in decathlon [67]. “Loss of motivation” represented one third of causes of dropouts [67]. However, the causes were determined by interview, without any validated tool or score, and no more psychological analyses.

##### 3.4.2.3 Environmental Aspects

Périard et al. [63] reported, in a retrospective study through questionnaire, exertional heat illness history, preparedness and recovery of athletes participated in the 2015 World Athletics Championships [63]. Among the 12 combined events athletes, the proportion of athletes who reported having experienced heat illness symptoms and diagnosis was higher than in other disciplines [63]. During 7 international outdoor Athletics championships, the lowest number of heat illnesses and illnesses per athlete were reported for combined events [102].

## 4 Discussion

The main findings of the present scoping review were that (1) a great number of articles provided information on performance and/or health in combined events, with balanced proportion of articles between performance and health, a variety of topics, and more than half focusing exclusively on understanding and/or analyzing combined events; (2) regarding performance, the majority of articles dealt with performance analysis/tactics/data management, followed by physiology, and nutrition; (3) regarding health, the majority of articles dealt with injuries, followed by physiology, illnesses, and nutrition; and (4) certain knowledge gaps were highlighted that can represent future research perspectives to better understand combined events, enhance performance and/or protect athletes' health.

### 4.1 Performance in Combined Events

#### 4.1.1 Current Knowledge on Performance in Combined Events

The present findings reported that the available scientific literature on combined events regarding performance provided (i) some evidence on *the who* are combined events athletes (e.g., body composition, maximal oxygen consumption, gene polymorphisms), (ii) lots of evidence on *the what* are the key events or key groups of events for performance in combined events, and (iii) some evidence on *the what* are the constraints/demands of a decathlon. The determinants of performance in combined events were mostly explored and understood through the analysis of performance data, in comparison to physiological, biomechanical or psychological experimentations. Analyses of performance data generally reported that speed, explosive strength, technique and endurance represented the main factors of performance in combined events [34, 68, 73, 74, 80, 81, 83, 85, 89, 96]. Some articles explored the physiological and biomechanical constraints during a competition of combined events: blood lactate [37], heart rate [48], blood glucose levels and food intakes [43], and power output [55], have been separately explored during a decathlon, and blood and urine markers of oxidative stress during a heptathlon [49]. Based on these findings, Edouard et al. [55] thus suggested that the decathlon cannot be compared to repeated‐sprint exercises, but rather to an intermittent‐maximal exercise [55]. Finally, in a qualitative analysis of the “most memorable decathlon,” Dale [33] reported that “when asked to describe their most memorable decathlon competition, each participant talked about a competition where he had the ability to overcome various problems and/or distractions to perform well” [33]. This can well represent the challenge of combined events performance: performing (or ability to perform) at best, in each event, over 1 or 2 days, with repetitive exposure to problems, distractors, and/or fatigue. Therefore, the combined events' Olympic champion is often referred to as the “World's greatest athlete” [33].

#### 4.1.2 Knowledge Gaps on Performance in Combined Event

This scoping review also highlighted important gaps in knowledge. In combined events athletes' characteristics, there was few information regarding physiological aspects related to respiratory, cardiovascular or musculoskeletal systems and functions. Except from score performance data analyses, there was very few information about performance determinants, for instance based on the physiological, biomechanical, technical, psychological capabilities. No articles dealt with *the how* to optimize combined events performance (e.g., training modalities, appropriate volumes, weekly organization of events, periodisation within the week, the season, the career, according to level and/or profiles). Some information about combined events training have been reported, however not in peer‐reviewed articles, presented as anecdotal experience of coaches (e.g., training experience of a Czech combined events group [129], decathlon general principles of training according to the time of the season in the United States of America [130], training for women heptathlon [131], combined events training for children and adolescents [4]). Except from the two articles by Kunz and Kaufmann [53, 54], no other article explored kinematics of combined events athletes, that could provide information regarding the potential increase in performance through improvement of the technique, and explanation about the performance‐related difference between combined events athletes and event‐specialist world class athletes (e.g., performance limitation through physiological or technical capabilities; are decathletes/heptathletes physically (strength, flexibility) able to reproduce the techniques that world class athletes of one events are doing?). Regarding nutrition and hydration, some data were available for women [40, 41], but very few for men [43], and it seems that current recommendations were not based on specific combined events data [28, 29, 32]. No articles dealt with psychological determinants of performance in combined events and only one article presented elements related to the “most memorable performance” [33]. Finally, there was very few information on performance in women (heptathlon or pentathlon), and none on decathlon for women [132].

### 4.2 Health in Combined Events

#### 4.2.1 Current Knowledge on Health in Combined Events

The present findings reported that the available scientific literature on combined events regarding health provided (i) lots of evidence on injuries in combined events during international Athletics championships in adult, (ii) few evidence on injuries during combined events competition for different levels or age groups, (iii) few evidence on injuries during a part, one or more seasons, (iv) few evidence on illnesses in combined events during international Athletics championships in adults, and (v) no information about other health‐related problems and pathologies. During adult international Athletics championships, combined events was the discipline with the highest number of injuries per 1000 registered athletes compared to all other Athletics disciplines [7]. Some events led to higher injury risk (i.e., pole vault and high jump for decathlon, and long jump for heptathlon) [116]. The characteristics of injuries in this specific context and by event has been described [3, 7, 116]. All articles about injuries corresponded to the steps 1 and 2 of the TRIPP framework (epidemiology and risk factors) [21], and there was no article on injury risk reduction strategies development, evaluation and implementation. Regarding illnesses, there were some information on illness incidence rates during championships, and their variation according to gender (higher rates in women than men) and according to type of championships (higher during outdoor than indoor championships) [8].

#### 4.2.2 Knowledge Gaps on Health in Combined Event

This scoping review also highlighted important gaps in knowledge. Apart from the overview of injuries during the 3–9 days of international adult championships, few or nothing is known about injuries during the other period of the Athletics season, illnesses during championships and during the season, specific short‐ and long‐term health‐related problems and pathologies usually explored in other sports (e.g., heart, respiratory, mental health) [14, 133, 134, 135, 136]. In addition, there was no article on injury and/or illness risk reduction strategies development, evaluation and implementation, and in general on health protection strategies for combined events athletes. Finally, no article reported or discussed the potential benefits of combined events participation for health.

### 4.3 Limitations

This scoping review has some limitations. Only one reviewer performed the articles selection and data extraction. No formal risk of bias assessment or critical appraisal of the methodological was conducted; this is however consistently with scoping reviews design [11]. The search through references lists of included articles found an important number of articles (*n* = 42). This can raise concerns about the quality of the database search and screening, and could also be explained by the non‐indexing in the usual databases of the journals in which these articles were published. Relevant articles may have been missed due to language restrictions or database limitation. The present scoping review included 5 narrative reviews [28, 29, 30, 31, 32] among the 111 included articles. The two narrative reviews on injuries in combined events (Edouard et al. [30] and Kim et al. [31]) were based on some articles included in the present scoping review, but published before 2016 and not all. The generalization of the current available scientific knowledge to all combined events athletes should be done with caution given some underrepresented populations. The author of the present scoping review is the first author or among the co‐authors of 26 (23.4%) articles, published since 2010, of the 111 articles included in the present study.

### 4.4 Perspectives for Research on Combined Events

The present findings highlighted the need for continuing high quality research on performance and/or health in combined events, preferably with a prospective design, including large sample sizes of combined events athletes (e.g., multicentric studies, international registries [116]), focused on underrepresented populations (e.g., women, adolescents, Masters athletes, athletes from other countries than the USA and France), in settings outside of the context of championships/competitions (e.g., over one or more Athletics season). Future studies should explore the potential influence or impact of the context [137], environmental conditions (e.g., weather: heat [102], cold [138], rain), or coaches [124, 139]. Qualitative approach should also be promoted, as did by Dale [33], to extend and deepen our understanding of combined events' challenges based on the perspectives of athletes, coaches, health professionals, or other stakeholders [137].

#### 4.4.1 Perspectives for Research on Performance in Combined Event

Future research should continue identifying the determinants of performance in combined events (i.e., *the what*), through exploring the specific physiological, biomechanical, psychological, contextual, environmental constraints and requirements of a competition in combined events (e.g., power, fatigue, kinetics, kinematics, stress, nutrition and hydration status). Research should also explore training modalities (i.e., *the how*) that allow to improve physiological, biomechanical, psychological characteristics and to achieve higher performance levels. There is also an interest to better understand the performance trade‐offs: how should training be distributed among weaker and stronger events so that athletes optimize their combined events' performance score?

#### 4.4.2 Perspectives for Research on Health in Combined Event

In general, future health research in combined event should consider a global, multi‐factorial, complex approach, including the biopsychosocial model [20]. Since combined events represent the Athletics discipline with the highest injury rate [7], there is a need to continue exploring in‐depth injury epidemiological outcomes and characteristics according to settings (e.g., training, competition), gender and age (e.g., youth and junior because of their immature musculoskeletal structures [97]). In addition, there is an interest to explore the injury consequences at long term, for instance after the career (e.g., osteoarthritis, chronic pain, disability) [136]. Efforts should also be made on the development of injury risk reduction approaches during the training preparation and in championships/competitions settings, considering the specificities of combined events. For illnesses, there is a need to determine illness epidemiological outcomes, characteristics and risk factors, during one or more Athletics season, according to settings (e.g., training, competition), gender and age, including for instance cardiovascular and respiratory diseases, relative energy deficiency in sport, mental health, use of medications and nutritional supplements [58], and lifestyle habits. Then, efforts should also be made to develop appropriate illness prevention strategies. Finally, it could be relevant to explore the health benefits‐risks balance in combined events.

### 4.5 Clinical Implications

For performance, it seems that all physical and physiological capabilities (e.g., speed, strength, flexibility, coordination and endurance) played a key role associated with technique [34, 68, 73, 80, 81, 85, 89, 96]. Consequently, it could be suggested to improve all these capabilities to improve combined events performance. For some aspects, technical events (e.g., pole vault, javelin, 110 m hurdles for decathlon, and long jump and javelin for heptathlon) seem to represent cornerstone of combined events performance [80, 89, 96], and should be the focus of training for physical and technical aspects. Since no scientific evidence is available to determine how to improve these capabilities and combined events performance, we have to refer to the experience of coaches. A coach of World Record holders, Olympic and World champions said that he “believes that success in the decathlon is based firstly on speed and then on strength and technique” and “technique changes with increasing speed and the development of strength,” meaning a continuing virtuous circle. Jerabek [4] reported that “a comprehensive and complex approach to combined events is the main principle on which the training must be built. Training for combined events cannot be just the sum of training means used in individual events (…). The training for the combined events will always involve compromise, between the development of apparently opposite movement skills and in how much time should be devoted to each of the individual disciplines. To complicate matters, these decisions must take into account the qualities, strengths and weaknesses of each individual athlete, so there is no universal scheme that can be applied” [4].

Combined events preparation should consider the available information on physiological and biomechanical constraints during a competition of combined events [37, 43, 48, 49, 55]. Following their results, Edouard et al. [55] suggested that an adapted wake‐up and warm‐up would be necessary and important when events take place in the morning, especially before the 110‐m hurdles, in order to improve performance, and from an injury risk reduction perspective. During competition, plyometric conditioning exercise (i.e., three 2‐legged rebound vertical jumps) executed 3 min before each attempt of long jump could be a relevant approach to maximize performance [56], with potential application also in other jumping events. Furthermore, coping with distractors during a competition could be another approach to maximize performance.

Pre‐participation health examination is recommended to screen for potential health‐related problems (e.g., cardiological problems, injuries) [140]. Based on the present scoping review, it seems relevant to add in such a pre‐participation health examination an evaluation of sleep [27] (e.g., with the Pittsburgh Sleep Quality Index (PSQI) [141] and/or Epworth Sleepiness Scale (ESS) [142]), energy availability [143], and postural control [61].

Given the notable number of injuries per registered athletes, efforts should be made to reduce injury risk. These strategies should target the most frequent injuries (i.e., located at the thigh, knee, ankle, and trunk, affected muscles, ligaments, skin and tendons) [7] and focus on the events with higher injury risk (i.e., pole vault and high jump for decathlon, and long jump for heptathlon) [116]. As there are, currently and to our knowledge, no injury risk reduction strategies scientifically validated in combined events, injury risk reduction strategies should follow general recommendations of following a multifactorial and multidimensional approach, considering biological/physical, psychological and societal/environmental aspects, inspiring from evidence from other Athletics disciplines and/or other sports [144, 145, 146, 147, 148, 149, 150, 151, 152].

## 5 Perspectives

Combined events are an Athletics discipline with specific and particular challenges for performance and health, supporting the interest of focused research on this discipline, despite concerning a small proportion of Athletics athletes [1]. The present systematic search found 111 articles providing data and information on performance and/or health in combined events. Their findings are of help to provide some clinical implications for performance enhancement (e.g., key events to orient training, competition management and coping) and health protection (e.g., pre‐participation health examination, injury risk reduction approach). Such an information can be of help to orient policy development and implementation, and promote the safety and well‐being of combined events. In addition, this methodological approach of an exhaustive summary and map of the available scientific literature on a sport could be applied to other sports to help practitioners and orient researchers. The present findings also highlighted the need for continuing research on performance and/or health in combined events. These future research should preferably be conducted using a prospective design, including large sample sizes of combined events athletes, focused on underrepresented populations, in settings outside of the context of championships/competitions, in order to extent the knowledge on *the who*, *the what* and *the how* for performance enhancement and health protection in a win‐win performance‐prevention approach.

## Author Contributions

P.E. conceived and developed the study; P.E. performed the data extraction and data analyses; P.E. interpreted the data, drafted the manuscript and approved the submitted manuscript. P.E. is the guarantor of the manuscript.

## Funding

The author has nothing to report.

## Disclosure

Equity, Diversity and Inclusion Statement: All relevant articles were included regardless of country, sex, age, participant level, ethnicity, socioeconomic status, or marginalized group representation. There was a unique author for this manuscript: a man, senior researcher, physician in physical medicine and rehabilitation and sports medicine and sports epidemiology, from France.

## Ethics Statement

The author has nothing to report.

## Conflicts of Interest

The author declares no conflicts of interest. P.E. is an Associate Editor for the British Journal of Sports Medicine, the BMJ Open Sports and Exercise Medicine, and the Scandinavian Journal of Medicine & Science in Sports.

## Supporting information


**Data S1:** Supporting Information

## Data Availability

Requests for data sharing from appropriate researchers and entities will be considered on a case‐by‐case basis. Interested parties should contact Pascal Edouard (pascal.edouard@univ-st-etienne.fr).
