# Supplementary material for: Performance and Health in Combined Events: A Scoping Review
Source: Scand J Med Sci Sports. 2025 Dec 31;36(1):e70190. doi: 10.1111/sms.70190 (PMC12755415; doi:10.1111/sms.70190)

ADDITIONAL ONLINE SUPPLEMENTAL MATERIAL:

Online supplemental Table 1. Research equation in the different databases: the MEDLINE (via PubMed), EMBASE (via Ovid), Web of Science (not applicable), and Google scholar databases, from inception to October 13, 2025.

The 13th October 2025

MEDLINE (via PubMed)	#1: “combined events”[All Fields] OR “decathlon”[All Fields] OR “heptathlon”[All Fields] OR “pentathlon” [All Fields] OR “decathlete*”[All Fields] OR “heptathlete*”[All Fields] OR “pentathlete*”[All Fields]<br>#2: “performance” OR “athletic performance” OR “sport performance” OR “physical performance” OR “training effects” OR “performance improvement” OR “performance enhancement” OR"physical fitness” OR “strength” OR “flexibility” OR “agility” OR “speed” OR “power” OR “endurance" OR “coordination” OR “balance” OR “technical skills” OR “technique” OR “technical performance” OR “skill” OR “skill development” OR “skill performance”<br>#3: “health*” OR “injur*” OR “illness*” OR “fitness” OR “morbidit*” OR “mortalit*” OR “well-being” OR “longevit*” OR “body composition*” OR “life expectanc*” OR “mental health” OR “wellness” OR “cardiovascular*”<br><br>#1 AND (#2 OR #3)<br>No filters were used in the electronic searches.	392 reports
EMBASE (via Ovid)	('combined events' OR 'decathlon' OR 'heptathlon' OR 'pentathlon' OR 'decathlete*' OR 'heptathlete*' OR 'pentathlete*') AND (('health*' OR 'illness*' OR 'injur*' OR 'fitness' OR 'morbidit*' OR 'mortalit*' OR 'well-being' OR 'longevit*' OR 'body composition*' OR 'life expectanc*' OR 'mental health' OR 'wellness' OR 'cardiovascular*') OR ('performance' OR 'athletic performance' OR 'sport performance' OR 'physical performance' OR 'training effects' OR 'performance improvement' OR 'performance enhancement' OR 'physical fitness' OR 'strength' OR 'flexibility' OR 'agility' OR 'speed' OR 'power' OR 'endurance' OR 'coordination' OR 'balance' OR 'technical skills' OR 'technique' OR 'technical performance' OR 'skill' OR 'skill development' OR 'skill performance'))	770 reports
Web of Science	#1 ("combined events"[All Fields] OR "decathlon"[All Fields] OR "heptathlon"[All Fields] OR "pentathlon" [All Fields] OR "decathlete*"[All Fields] OR "heptathlete*"[All Fields] OR "pentathlete*"[All Fields])<br>#2 ("performance" OR "athletic performance" OR "sport performance" OR "physical performance" OR "training effects" OR "performance improvement" OR "performance enhancement" OR "physical fitness" OR "strength" OR "flexibility" OR "agility" OR "speed" OR "power" OR "endurance" OR "coordination" OR "balance" OR "technical	40 reports

	skills" OR "technique" OR "technical performance" OR "skill" OR "skill development" OR "skill performance") (All Fields)<br>#3 ("health*" OR "illness*" OR "injur*" OR "fitness" OR "morbidit*" OR "mortalit*" OR "well-being" OR "longevit*" OR "body composition*" OR "life expectanc*" OR "mental health" OR "wellness" OR "cardiovascular*") (All Fields)<br>#1 AND (#2 OR #3)	
Google scholar	(“combined events”[All Fields] OR “decathlon”[All Fields] OR “heptathlon”[All Fields] OR “pentathlon” [All Fields] OR “decathlete*”[All Fields] OR “heptathlete*”[All Fields] OR “pentathlete*”[All Fields]) AND ((“performance” OR “athletic performance” OR “sport performance” OR “physical performance” OR “training effects” OR “performance improvement” OR “performance enhancement” OR “physical fitness” OR “strength” OR “flexibility” OR “agility” OR “speed” OR “power” OR “endurance" OR “coordination” OR “balance” OR “technical skills” OR “technique” OR “technical performance” OR “skill” OR “skill development” OR “skill performance”) OR (“health*” OR “injur*” OR “illness*” OR “fitness” OR “morbidit*” OR “mortalit*” OR “well-being” OR “longevit*” OR “body composition*” OR “life expectanc*” OR “mental health” OR “wellness” OR “cardiovascular*”))	1 report

Online supplemental Table 2. List of the 5 reports excluded because the full texts were not retrieved, and of the 31 reports based on the full text screening with the respective reasons.

Articles	Reasons for exclusion
Bradshaw EJ, Aisbett B. Visual guidance during competition performance and run-through training in long jumping. Sports Biomechanics. 2006;5(1): 1-14	No specific data on combined events
Casali N, Ghisi M, Jansen P, Feraco T, Meneghetti C. What Can Affect Competition Anxiety in Athletes? The Role of Self-Compassion and Repetitive Negative Thinking. Psychological Reports. 2022; 125(4): 2009-2028	No specific data on combined events
Conti A, Rosponi A, Dapretto L, Magini V, Felici F. Cardiac and metabolic demands of in place shallow water running in trained and untrained men. J Sports Med Phys Fitness. 2008;48(2): 182-189	Not on combined events
Edouard P, Caumeil B, Giroux C, Bruneau A, Tondut J, Navarro L, Hanon C, Guilhem G, Ruffault A. Epidemiology of Injury Complaints in Elite Sprinting Athletes in Athletics (Track and Field). Applied Science. 2023;13(14): 8105	No specific data on combined events
Edouard P, Depiesse F, Alonso JM, Branco P. Injury and illness risks during outdoor European athletics championships: Analysis of Helsinki 2012 championships. Annals of Physical and Rehabilitation Medicine. 2014;57(1): e270	Congress abstract
Edouard P, Morin JB, Samozino P. Changes of maximal power output of lower extremity during a decathlon. Annals of Physical and Rehabilitation Medicine. 2014;57(1): e327	Congress abstract
Edouard P, Serra JM, Hertert P, Cugy E, Morel N, Prevost M, Depiesse F. Athletic injury prevention: Epidemiology of injuries during the French Elite championships. Annals of Physical and Rehabilitation Medicine. 2016;56(Supp): e21	Congress abstract
Fer C, Serra JM, Depiesse F, Edouard P. Analyse de l'efficacité du programme « Décathlon de prévention des blessures » en athlétisme : étude pilote de faisabilité. Journal de Traumatologie du Sport. 2017;34(1): 15-28	No specific data on combined events
Fu P. Research on the Development Trend of Decathlon Athletics Performance in Liaoning Province. Proceedings of the 2nd International Conference on Contemporary Education, Social Sciences and Humanities (ICCESSH 2017). 2017. DOI: 10.2991/iccessh-17.2017.229	Congress abstract
Garzon FC. Nonclassical connectionism should enter the decathlon. Behavioral and Brain Sciences. 2003;26(5): 603-604	Not on combined events
Gumus E, Akgul S, Kanbur N, Derman O. A comparison of bone mineral density in adolescent swimmers, pentathletes and figure skaters. Turk J Pediatr. 2019;61(6): 831-838	Not on combined events
Iellamo F, Pigozzi F, Parisi A, Di Salvo V, Vago T, Norbiato G, Lucini D, Pagani M. The stress of competition dissociates neural and cortisol homeostasis in elite athletes. J Sports Med Phys Fitness. 2003;43(4): 539-545	Not on combined events
Junge A, Engebretsen L, Mountjoy M, Alonso JM, Renstrom P, Aubry AJ, Dvorak J. Sports injuries during the Summer Olympic Games 2008. Am J Sports Med. 2009;37(11): 2165-2172	No specific data on combined events
Kalabiska I, Zsakai A, Annar D, Malina RM, Szabo T. Sport Activity Load and Skeletomuscular Robustness in Elite Youth Athletes. Int J Environ Res Public Health. 2022;19(9): 5083	Not on combined events
Lepore L, Francobandiera C, Maffulli N. Fracture of the Os tibiale externum in a decathlete J Foot Surg. 1990;29(4): 366-368	No full text available
Lhee SH, Jain R, Sadasivam MM, Kim S, Bae M, Yu J, Lee DY. Sports injury and illness incidence among South Korean elite athletes in the 2018 Asian Games/ a single- physician prospective study of 782 athletes. BMJ Open Sport Exerc Med. 2021;7(1): e000689	No specific data on combined events
Li G, Boncz I, Járomi M, Molics B, Ács P, Tardi P. 01Survey of Sport-Specific Lower Limb Injuries in 15 of Hungary's Most Popular Sport. Value Health. 2022;25(12): S496-S497	Congress abstract

Logue DM, Mahony L, Corish CA, Tobin D, Doherty R, O'Higgins G, Madigan SM. Athletes' and Coaches' Perceptions of Nutritional Advice: Eating More Food for Health and Performance. Nutrients. 2021;13(6): 1965	Not on combined events
Ma X, Practical research on promoting the training of excellent music professional talents by the pentathlon competition. Basic Clin Pharmacol Toxicol. 2019;125: 64-65	Not on combined events
Martinez-Silvan D, Johnson A, Tramullas A. Skeletal maturity as injury risk factor in adolescent elite track and field athletes. The Orthopaedic Journal of Sports Medicine. 2018;6(6): DOI: 10.1177/2325967118S00038	Congress abstract
Millet GP, Brocherie F, Burtscher J. Olympic Sports Science - Bibliometric Analysis of All Summer and Winter Olympic Sports Research. Front Sports Act Living. 2021;3:772140. doi:10.3389/fspor.2021.77214	No specific data on combined events
Onorato EA, Grancini L, Monizzi G, Mastrangelo A, Fabbiocchi F, Bartorelli AL. Noblestitch® system for PFO closure/ A novel but judicious alternative to traditional devices: A case report. Front Cardiovascular Med. 2023:10:1095661	Not on combined events
Parnat J, Viru A, Savi T. Untersuchungen der aeroben und anaeroben leistungsfahigkeit von zehnkampfern. 1973;13(12): 366-369	No full text available
Piccininni JJ, Drover JM. Ankle sprain in an elite decathlete. Athl Ther Today. 2003;8(2): 26-27	No full text available
Pokrywka A, Berezovska D, Lewandowska-Pachecka S, Rynkowski M, Faiss R, Krzywanski J. Evaluation of the use of glucocorticosteroids by athletes in Poland in the light of the amended anti-doping regulations. Farmacja Polska. 2022;78(1): 3-9	Other languages
Prendergast H, Bannen T, Erickson T, Honore KR. The toxic torch of modern Olypic Games. Vet Hum Toxicol. 2003;45(2): 97-102	No full text available
Purdy JG, Computer generated track and field scoring tables/ I. Historical development. Med Sci Sports. 1974;6(4):287-294	No specific data on combined events
Resch M, Haasz P. The first epidemiology survey among Hungarian elite athletes-Eating disorders, depression and risk factors. Orvosi Hetilap. 2009;150(1): 35-40	Other languages
Rubal BJ, Moody JM, Damore S, Bunker SR, Diaz NM. Left ventricular performance of the athletic heart during upright exercise/ a heart rate-controlled study. Medicine and Science in Sports and Exercise. 1986;18(1): 134-140	Not on combined events
Rubal BJ, Moody JM, Damore S, Al-Muhailani AR. Heart Size and Function ofSoldiers, Athletes, and Sedentary Men. Military Medicine. 1989;154(3): 153-158	Not on combined events
Schwanz K, McInnis K. Searching for a Javelin in a Haystack: Uncommon Cause of Chest Wall Pain in a Division 1 Heptathlete. Clin J Sport Med. 2025;35(3): e22-e23	Congress abstract
Sinyavskiy YA, Tuigunov DN, Kapysheva UN, Bakhtiyarova SK, Sarsembayev KS, Zhunussova GS, Omarov YN, Bekmanov BO, Zhaksymov BI, Makashev EE, Junussova AB. [The effect of intaking a new sport food on milk and fruit basis on blood biochemical indicators and antioxidant status in athletes]. Vopr Pitan. 2024;93(5): 43-56. doi: 10.33029/0042-8833	No full text available
Sydo N, Emese C, Szigyártó I, Kaufmann M, Lakatos BK, Kovács A, Bárczi G, Czimbalmos C, Vágó H, Becker D, Allison TG, Merkely B. Repetitive syncope during pentathlon competition. J. Am. Coll. Cardiol. 2020;75(11): 3065	Congress abstract
Timpka T, Périard JD, Spreco A, Dahlström Ö, Jacobsson J, Bargoria V, Andersson C, Alonso J-M, Racinais S. Health complaints and heat stress prevention strategies during taper as predictors of peaked athletic performance at the 2015 World Athletics Championship in hot conditions. J. Sci. Med. Sport. 2020;23(4): 336-341	No specific data on combined events
Yoshitake R, Shrestha M, Ogata H, Nagayama H, Omi N. Energy Balance During Competition in the Heptathlon. Int J Sport Nutr Exer Metabol. 2022;32(0): S4-S5	Congress abstract
Anthropometric Characteristics of Elite and Olympic Mexican Track and Field Athletes (ANTHROLYMPMX) - Registration protocol. 2024. DOI: 10.13140/RG.2.2.27289.63847	No peer-review

Online supplemental Table 3: Characteristics of the 111 included articles.

Authors and years	Articles focus on Combined events understanding	Type of Research	Type of articles	Research approach (quantitative or qualitative)	Study design approach (Descriptive vs. Analytic)	Study design	Level of evidence	N	Sex (W/M)	Country	Performance	Health
Aguilar-Navarro et al. 2021[1]	No	Primary	Original research	Quantitative	Descriptive	Retrospective	2b	NR	NR/NR	International	No	Yes
Allen et al. 2025[2]	No	Primary	Case report	Quantitative	Descriptive	Prospective	4	1	1/0	USA	No	Yes
Alonso et al. 2009[3]	No	Primary	Original research	Quantitative	Descriptive	Prospective	1b	69	39/30	International	No	Yes
Alonso et la. 2010[4]	No	Primary	Original research	Quantitative	Descriptive	Prospective	1b	70	29/41	International	No	Yes
Alonso et al. 2012[5]	No	Primary	Original research	Quantitative	Descriptive	Prospective	1b	59	29/30	International	No	Yes
Alonso et al. 2015[6]	No	Primary	Original research	Quantitative	Analytic	Prospective	1b	23	NR/NR	International	No	Yes
Anand et al. 2012[7]	No	Primary	Case report	Quantitative	Descriptive	Prospective	4	1	0/1	UK	No	Yes
Ashino et al. 2023[8]	Yes	Primary	Original research	Quantitative	Analytic	Retrospective	2b	807	0/807	Japan	Yes	No
Battles et al. 2025[9]	Yes	Primary	Original research	Quantitative	Analytic	Retrospective	2b	642	0/642	International	Yes	No
Beaulieu et al. 1995[10]	Yes	Primary	Original research	Quantitative	Descriptive	Prospective	4	7	0/7	France	Yes	Yes
Ben-Zaken et al. 2022[11]	Yes	Primary	Original research	Quantitative	Descriptive	Cross-sectional	2b	27	0/27	Israel and Estonia	Yes	No
Bennell et al. 1996 [12]	No	Primary	Original research	Quantitative	Descriptive	Prospective	1b	4	4/0	Australia	No	Yes
Bilic 2015[13]	Yes	Primary	Original research	Quantitative	Analytic	Retrospective	2b	450	0/450	International	Yes	No
Bilic et al. 2012[14]	Yes	Primary	Original research	Quantitative	Analytic	Retrospective	2b	1	1/0	Germany	Yes	No
Bilic et al. 2015[15]	Yes	Primary	Original research	Quantitative	Analytic	Retrospective	2b	450	0/450	International	Yes	No
Bilic et al. 2015[16]	Yes	Primary	Original research	Quantitative	Analytic	Retrospective	2b	100	0/100	International	Yes	No
Bogdanis et al. 2017[17]	No	Primary	Original research	Quantitative	Analytic	Prospective	2b	8	0/8	Greece	Yes	No
Bowman etal. 2008[18]	No	Primary	Case report	Quantitative	Descriptive	Prospective	4	1	0/1	USA	No	Yes
Brodani et al. 2020[19]	Yes	Primary	Original research	Quantitative	Analytic	Retrospective	2b	255	0/255	International	Yes	No
Canda Moreno et al. 2001[20]	Yes	Primary	Original research	Quantitative	Descriptive	Cross-sectional	3	9	0/9	Spain	Yes	Yes
Careau et al. 2017[21]	Yes	Primary	Original research	Quantitative	Analytic	Retrospective	2b	1369	733/636	International	Yes	No

Castlereagh et al. 2005[22]	No	Primary	Case report	Quantitative	Descriptive	Prospective	4	1	1/0	Australia	No	Yes
Chapon et al. 2022[23]	No	Primary	Original research	Quantitative	Analytic	Retrospective	2b	4	1/3	France	Yes	Yes
Chatterjee et al. 1991[24]	No	Primary	Original research	Quantitative	Descriptive	Cross-sectional	3	3	3/0	India	Yes	Yes
Cox et al. 2002[25]	Yes	Primary	Original research	Quantitative	Analytic	Retrospective	2b	NR	0/NR	International	Yes	No
Cuddeford et al. 2020[26]	No	Primary	Case report	Quantitative	Descriptive	Prospective	4	2	0/2	USA	No	Yes
D'Souza 1994[27]	No	Primary	Original research	Quantitative	Descriptive	Retrospective	2b	6	NR/NR	UK	No	Yes
Dale 2000[28]	Yes	Primary	Original research	Qualitative	Descriptive	Prospective	1b	7	0/7	USA	Yes	No
Dawkins et al. 1994[29]	Yes	Primary	Original research	Quantitative	Analytic	Retrospective	2b	26	26/0	International	Yes	No
Dinnie et al. 2020[30]	Yes	Primary	Original research	Quantitative	Analytic	Retrospective	2b	155	155/0	International	Yes	No
Durand et al. 1996[31]	Yes	Primary	Original research	Quantitative	Descriptive	Prospective	4	6	0/6	France	Yes	Yes
Dziadek et al. 2016[32]	Yes	Primary	Original research	Quantitative	Analytic	Retrospective	2b	25	0/25	International	Yes	No
Dziadek et al. 2018[33]	Yes	Primary	Original research	Quantitative	Analytic	Retrospective	2b	25	0/25	Poland	Yes	No
Dziadek et al. 2022[34]	Yes	Primary	Original research	Quantitative	Analytic	Retrospective	2b	25	0/25	International	Yes	No
Edouard 2011[35]	Yes	Primary	Original research	Quantitative	Descriptive	Retrospective	2b	2111	0/2111	International	Yes	No
Edouard et al. 2010[36]	Yes	Primary	Original research	Quantitative	Descriptive	Prospective	2b	50	0/50	France	Yes	Yes
Edouard et al. 2012[37]	Yes	Primary	Original research	Quantitative	Descriptive	Prospective	1b	107	48/59	France	Yes	Yes
Edouard et al. 2012[38]	Yes	Primary	Original research	Quantitative	Descriptive	Retrospective	2b	69	30/39	France	No	Yes
Edouard et al. 2013[39]	No	Primary	Original research	Quantitative	Descriptive	Prospective	1b	31	15/16	Europe	No	Yes
Edouard et al. 2014[40]	No	Primary	Original research	Quantitative	Descriptive	Prospective	1b	50	23/27	Europe	No	Yes
Edouard et al. 2014[41]	No	Primary	Original research	Quantitative	Descriptive	Prospective	1b	111	NR/NR	International	No	Yes
Edouard et al. 2015[42]	No	Primary	Original research	Quantitative	Descriptive	Prospective	1b	NR	NR/NR	International	No	Yes
Edouard et al. 2015[43]	Yes	Primary	Original research	Quantitative	Descriptive	Prospective	4	6	0/6	France	Yes	No
Edouard et al. 2016[44]	No	Primary	Original research	Quantitative	Descriptive	Prospective	1b	NR	NR/NR	International	No	Yes
Edouard et al. 2016[45]	No	Secondary	Narrative review	NA	Descriptive	NA	5	NA	NA/NA	NA	No	Yes
Edouard et al. 2019[46]	No	Primary	Original research	Quantitative	Descriptive	Prospective	1b	478	224/254	International	No	Yes
Edouard et al. 2020[47]	No	Primary	Original research	Quantitative	Descriptive	Prospective	1b	635	307/328	International	No	Yes
Edouard et al. 2021[48]	Yes	Primary	Original research	Quantitative	Analytic	Prospective	1b	286	141/145	International	Yes	Yes

Edouard et al. 2021[49]	No	Primary	Original research	Quantitative	Descriptive	Prospective	1b	NR	NR/NR	International	No	Yes
Edouard et al. 2022[50]	No	Primary	Original research	Quantitative	Descriptive	Retrospective	2b	13	8/5	Europe	Yes	Yes
Edouard et al. 2022[51]	No	Primary	Original research	Quantitative	Analytic	Prospective	1b	44	21/23	International	No	Yes
Edouard et al. 2024[52]	No	Primary	Original research	Quantitative	Descriptive	Retrospective	2b	51	NR/NR	France	Yes	Yes
Edouard et al. 2024[53]	No	Primary	Original research	Quantitative	Descriptive	Cross-sectional	2b	154	NR/NR	France	No	Yes
Edouard et al. 2024[54]	No	Primary	Original research	Quantitative	Descriptive	Retrospective	2b	NR	NR/NR	France	No	Yes
Edouard et al. 2025[55]	Yes	Primary	Original research	Quantitative	Descriptive	Prospective	1b	621	307/314	International	No	Yes
Enoki et al. 2021[56]	Yes	Primary	Original research	Quantitative	Descriptive	Prospective	1b	8	0/8	Japan	No	Yes
Fan 2014[57]	Yes	Primary	Original research	Quantitative	Analytic	Retrospective	2b	28	0/28	China	Yes	No
Faris et al. 1980[58]	Yes	Primary	Original research	Quantitative	Descriptive	Cross-sectional	3	10	0/10	USA	Yes	Yes
Fedderman et al. 2014[59]	No	Primary	Original research	Quantitative	Descriptive	Prospective	1b	616	NR/NR	International	No	Yes
Fisher et al. 1989[60]	Yes	Primary	Original research	Quantitative	Descriptive	Cross-sectional	3	4	0/4	USA	Yes	Yes
Ganse et al. 2014[61]	No	Primary	Original research	Quantitative	Descriptive	Prospective	1b	498	NR/NR	Europe	No	Yes
Garcia-Roca et al. 2022[62]	Yes	Primary	Original research	Quantitative	Analytic	Retrospective	2b	102	0/102	International	Yes	No
Gassmann et al. 2016[63]	Yes	Primary	Original research	Quantitative	Analytic	Retrospective	2b	10	10/0	International	Yes	No
Gregor et al. 1981[64]	No	Primary	Original research	Quantitative	Descriptive	Cross-sectional	3	6	6/0	USA	Yes	Yes
Hiroshige et al. 2025[65]	No	Primary	Original research	Quantitative	Descriptive	Retrospective	2b	438	229/209	Japan	No	Yes
Hollander et al. 2021[66]	No	Primary	Original research	Quantitative	Descriptive	Prospective	1b	NR	NR/NR	International	No	Yes
Horst et al. 2020[67]	Yes	Primary	Original research	Quantitative	Descriptive	Cross-sectional	3	7	0/7	Germany	Yes	No
Houtkooper et al. 2001[68]	Yes	Primary	Original research	Quantitative	Descriptive	Cross-sectional	3	19	19/0	USA	Yes	Yes
Houtkooper et al. 2007[69]	No	Secondary	Narrative review	NA	NA	NA	5	NA	NA/NA	NA	Yes	Yes
Jacobsson et al. 2012[70]	No	Primary	Original research	Quantitative	Descriptive	Cross-sectional	2b	17	12/5	Sweden	No	Yes
Jacobsson et al. 2013[71]	No	Primary	Original research	Quantitative	Descriptive	Prospective	1b	23	15/8	Sweden	No	Yes
Jin et al. 2014[72]	Yes	Primary	Original research	Quantitative	Descriptive	Retrospective	2b	13	13/0	International	Yes	No
Karvonen et al. 1953[73]	No	Primary	Original research	Quantitative	Analytic	Retrospective	2b	62	0/62	International	Yes	No
Kenny et al. 2005[74]	Yes	Primary	Original research	Quantitative	Analytic	Retrospective	2b	92	0/92	International	Yes	No

Kim et al. 2016[75]	Yes	Secondary	Narrative review	Quantitative	Descriptive	NA	5	NA	NA/NA	NA	No	Yes
Krahenbuhl et al. 1979[76]	Yes	Primary	Original research	Quantitative	Descriptive	Cross-sectional	3	9	9/0	USA	Yes	Yes
Kunz et al. 1981[77]	Yes	Primary	Original research	Quantitative	Analytic	Cross-sectional	3	16	0/16	Switzerland	Yes	No
Kunz et al. 1983[78]	Yes	Primary	Original research	Quantitative	Analytic	Cross-sectional	3	12	0/12	Switzerland	Yes	No
Leonhardt et al. 2024[79]	No	Primary	Original research	Quantitative	Descriptive	Cross-sectional	3	3	NR/NR	International	Yes	Yes
Linden 1977[80]	Yes	Primary	Original research	Quantitative	Analytic	Retrospective	2b	139	0/139	International	Yes	No
Maimoun et al. 2008[81]	No	Primary	Original research	Quantitative	Descriptive	Cross-sectional	3	13	0/13	France	No	Yes
Mayr et al. 1988[82]	Yes	Primary	Original research	Quantitative	Descriptive	Retrospective	2b	71	0/71	Germany	No	Yes
Mullins et al. 2001[83]	Yes	Primary	Original research	Quantitative	Descriptive	Cross-sectional	3	19	19/0	USA	Yes	Yes
Nunes et al. 2025[84]	Yes	Primary	Original research	Quantitative	Descriptive	Cross-sectional	3	31	0/31	Brazil	Yes	No
Palamarchuk et al. 2000[85]	No	Primary	Case report	Quantitative	Descriptive	Prospective	4	1	1/0	USA	No	Yes
Panday et al. 2019[86]	Yes	Primary	Original research	Quantitative	Descriptive	Retrospective	2b	446	0/446	International	Yes	No
Park et al. 2011[87]	Yes	Primary	Original research	Quantitative	Analytic	Retrospective	2b	166	0/166	International	Yes	No
Pastre et al. 2005[88]	No	Primary	Original research	Quantitative	Descriptive	Retrospective	2b	NR	NR/NR	Brazil	No	Yes
Pavlovic 2017[89]	Yes	Primary	Original research	Quantitative	Analytic	Retrospective	2b	10	0/10	International	Yes	No
Pavlovic et al. 2020[90]	Yes	Primary	Original research	Quantitative	Analytic	Retrospective	2b	5	0/5	International	Yes	No
Périard et al. 2017[91]	No	Primary	Original research	Quantitative	Descriptive	Retrospective	2b	12	4/8	International	Yes	Yes
Purdy et al. 1976[92]	Yes	Primary	Original research	Quantitative	Descriptive	Prospective	1b	11	0/11	USA	Yes	No
Remmel et al. 2023[93]	Yes	Primary	Original research	Quantitative	Descriptive	Cross-sectional	3	27	0/27	Europe	Yes	No
Reuter et al. 2017[94]	Yes	Primary	Original research	Quantitative	Analytic	Cross-sectional	3	8	0/8	Germany	Yes	Yes
Ruff 2000[95]	No	Primary	Original research	Quantitative	Analytic	Retrospective	2b	12	11/1	USA	Yes	Yes
Samia et al. 2014[96]	Yes	Primary	Original research	Quantitative	Descriptive	Prospective	1b	8	8/0	Egypt	Yes	Yes
Schomaker et al. 2011[97]	Yes	Primary	Original research	Quantitative	Analytic	Retrospective	2b	30	0/30	International	Yes	No
Shirreffs et al. 2007[98]	No	Secondary	Narrative review	NA	NA	NA	5	NA	NA/NA	NA	Yes	Yes
Sygo et al. 2019[99]	No	Secondary	Narrative review	NA	NA	NA	5	NA	NA/NA	NA	Yes	Yes

Tscholl et al. 2010[100]	No	Primary	Original research	Quantitative	Descriptive	Retrospective	2b	122	NR/NR	International	No	Yes
Van Damme et al. 2002[101]	Yes	Primary	Original research	Quantitative	Analytic	Retrospective	2b	600	0/600	International	Yes	No
Walker et al. 2015[102]	Yes	Primary	Original research	Quantitative	Analytic	Retrospective	2b	611	0/611	International	Yes	No
Wang et al. 2007[103]	Yes	Primary	Original research	Quantitative	Descriptive	Retrospective	2b	8	0/8	Czech	Yes	No
Ward et al. 2002[104]	Yes	Primary	Original research	Quantitative	Analytic	Retrospective	2b	NR	0/NR	International	Yes	No
Wentz et al. 1997[105]	Yes	Primary	Original research	Quantitative	Descriptive	Retrospective	2b	50	0/50	Germany	No	Yes
Wimmer et al. 2011[106]	Yes	Primary	Original research	Quantitative	Analytic	Retrospective	2b	3103	0/3103	International	Yes	No
Withers et al. 1987[107]	No	Primary	Original research	Quantitative	Descriptive	Cross-sectional	3	3	0/3	Australia	Yes	Yes
Woolf et al. 2007[108]	Yes	Primary	Original research	Quantitative	Analytic	Retrospective	2b	173	0/173	International	Yes	No
Yoshitake et al. 2024[109]	Yes	Primary	Original research	Quantitative	Descriptive	Prospective	4	10	0/10	Japan	Yes	Yes
Zhang et al. 2014 [110]	Yes	Primary	Original research	Quantitative	Analytic	Retrospective	2b	16	16/0	International	Yes	No
Zhang et al. 2014[111]	Yes	Primary	Original research	Quantitative	Analytic	Retrospective	2b	28	0/28	International	Yes	No

N: Number; W: Women; M: Men; NA: Not applicable; NR: Not reported; USA: United States of America; UK: United Kingdom.

References:

1 Aguilar-Navarro M, Salinero JJ, Muñoz-Guerra J, *et al.* Frequency and type of adverse analytical findings in athletics: Differences among disciplines. *Drug Test Anal*. 2021;13:1561–8. doi: 10.1002/dta.3058

2 Allen SMF, Bartaczewicz BL, Molenhouse AE, *et al.* Don't Sleep on Sleep: A Clinical CASE Report From a Division I Heptathlete. *J Athl Train*. 2025;60:296–300. doi: 10.4085/1062-6050-0537.24

3 Alonso JM, Junge A, Renström P, *et al.* Sports injuries surveillance during the 2007 IAAF world athletics championships. *Clinical Journal of Sport Medicine*. 2009;19:26–32. doi: 10.1097/JSM.0b013e318191c8e7

4 Alonso JM, Tscholl PM, Engebretsen L, *et al.* Occurrence of injuries and illnesses during the 2009 IAAF World Athletics Championships. *Br J Sports Med*. 2010;44:1100–5. doi: 10.1136/bjsm.2010.078030

5 Alonso JM, Edouard P, Fischetto G, *et al.* Determination of future prevention strategies in elite track and field: Analysis of Daegu 2011 IAAF Championships injuries and illnesses surveillance. *Br J Sports Med*. 2012;46:505–14. doi: 10.1136/bjsports-2012-091008

6 Alonso J-M, Jacobsson J, Timpka T, *et al.* Preparticipation injury complaint is a risk factor for injury: A prospective study of the Moscow 2013 IAAF Championships. *Br J Sports Med*. 2015;49:1118–24. doi: 10.1136/bjsports-2014-094359
7 Anand A, Campion NJ, Chakraverty R, *et al.* Bilateral snapping ulnar nerve syndrome in a professional decathlete-a rare case report and review of the literature. *Eur Orthop Traumatol*. 2012;3:263–6. doi: 10.1007/s12570-012-0111-0
8 Ashino Y, Ikuta Y, Kida N. Estimation of factors contributing to level differences in Japanese university decathlon athletes. *PLoS One*. 2023;18:e0295083. doi: 10.1371/journal.pone.0295083
9 Battles P, Noble TJ, Chapman RF. Predicting high-performance decathlon career best. *Exp Physiol*. Published Online First: 2025. doi: 10.1113/EP091921
10 Beaulieu P, Ottoz H, Grange C, *et al.* Blood lactate levels of decathletes during competition. *Br J Sports Med*. 1995;29:80–4.
11 Ben-Zaken S, Meckel Y, Remmel L, *et al.* The prevalence of IGF-I axis genetic polymorphisms among decathlon athletes. *Growth Hormone and IGF Research*. 2022;64:101468. doi: 10.1016/j.ghir.2022.101468
12 Bennell KL, Malcolm SA, Thomas SA, *et al.* The incidence and distribution of stress fractures in competitive track and field athletes: A twelve-month prospective study. *Am J Sports Med*. 1996;24:211–7. doi: 10.1177/036354659602400217
13 Bilić M. Determination of taxonomic type structures of top decathlon athletes. *Acta Kinesiologica*. 2015;9:20–3.
14 Bilic M, Smajlovic N. Model for longitudinal analysis of an individual all-rounder athlete's potential. *Homosporticus*. 2012;44–8.
15 Bilić M, Smajlović N, Balic A. Contribution to discipline decathlon total score results in relation to decathlon age and result level. *Acta Kinesiologica*. 2015;9:66–9.
16 Bilić M, Smajlović N. Real and hypothetical maximum potential scale ranges of most successful world decathlons. *Acta Kinesiologica*. 2015;9:88–91.
17 Bogdanis GC, Tsoukos A, Veligekas P. Improvement of long-jump performance during competition using a plyometric exercise. *Int J Sports Physiol Perform*. 2017;12:235–40. doi: 10.1123/ijspp.2016-0116
18 Bowman TG, Palmieri-Smith R. Proximal Humeral Epiphyseal Plate Fracture in a Collegiate Track and Field Decathlete: A Case Study. *J Sport Rehabil*. 2008;17:76–83.
19 Brodáni J, Dvořáčková N, Czaková M. Classification of athletic decathlon using methods of hierarchical analysis. *Journal of Physical Education and Sport*. 2020;20:3253–9. doi: 10.7752/jpes.2020.s6441
20 Canda Moreno A, Sainz Fernandez L, De Diego Lobo T, *et al.* Morphological characteristics of the decathlete versus specialists. *Achirvos de medicina del deporte*. 2001;18:277–84.
21 Careau V, Wilson RS. Performance trade-offs and ageing in the 'world's greatest athletes'. *Proceedings of the Royal Society B: Biological Sciences*. 2017;284. doi: 10.1098/rspb.2017.1048
22 Castlereagh F, Pollard H. Traumatic spondylolysis in a heptathlete: a case history and review. *J Chiropr Med*. 2005;4:89–96.

23 Chapon J, Navarro L, Edouard P. Relationships Between Performance and Injury Occurrence in Athletics (Track and Field): A Pilot Study on 8 National-Level Athletes From Sprints, Jumps and Combined Events Followed During at Least Five Consecutive Seasons. *Front Sports Act Living*. 2022;4:852062. doi: 10.3389/fspor.2022.852062
24 Chatterjee S, Saha SK, Saha D, *et al.* Maximal aerobic capacity of Bengali girl athletes of different sports activities. *Japanese Journal of Physiology*. 1991;41:397–411.
25 Cox TF, Dunn RT. An Analysis of Decathlon Data. *Journal of the Royal Statistical Society Series D (The Statistician)*. 2002;51:179–87.
26 Cuddeford T, Brumitt J. In-season rehabilitation program using blood flow restriction therapy for two decathletes with patellar tendinopathy: a case report. *Int J Sports Phys Ther*. 2020;15:1184–95. doi: 10.26603/ijspt20201184
27 D'Souza D. Track and Field athletics injuries -- a one-year survey*. *Br J Sports Med*. 1994;28:197–202.
28 Dale GA. Distractions and coping strategies of elite decathletes during their most memorable performances. *Sport Psychol*. 2000;14:17–41.
29 Dawkins BP, Andreae PM, O'Connor PM. Analysis of Olympic Heptathlon Data. *J Am Stat Assoc*. 1994;89:1100–6.
30 Dinnie A, O'Donoghue P. Strategic target setting in the heptathlon. *Journal of Sports Analytics*. 2020;6:129–45. doi: 10.3233/jsa-200351
31 Durand S, Beaune B. Variation de la fréquence cardiaque au cours du décathlon. *Sci Sports*. 2006;21:32–5. doi: 10.1016/j.scispo.2005.11.003
32 Dziadek B, Iskra J, Przednowek K. Running preparation and the final decathlon score in terms of sports career development. *Physical Activity Review*. 2016;4:115–23. doi: 10.16926/par.2016.04.14
33 Dziadek B, Iskra J, Przednowek K. Principal Component Analysis in the Study of Structure of the Best Polish Decathlon Competitors from the Period between 1985–2015. *Central European Journal of Sport Sciences and Medicine*. 2018;23:77–87. doi: 10.18276/cej.2018.3-08
34 Dziadek B, Iskra J, Mendyka W, *et al.* Principal component analysis in the study of the structure of decathlon at different stages of sports career. *Polish Journal of Sport and Tourism*. 2022;29:21–8. doi: 10.2478/pjst-2022-0023
35 Edouard P. Frequency of dropouts in decathlon: An epidemiological retrospective study. *Sci Sports*. 2011;26:97–100. doi: 10.1016/j.scispo.2010.11.002
36 Edouard P, Pruvost J, Edouard JL, *et al.* Causes of dropouts in decathlon. A pilot study. *Physical Therapy in Sport*. 2010;11:133–5. doi: 10.1016/j.ptsp.2010.07.004
37 Edouard P, Samozino P, Escudier G, *et al.* Injuries in youth and national combined events championships. *Int J Sports Med*. 2012;33:824–8. doi: 10.1055/s-0031-1301332
38 Edouard P, Kerspern A, Pruvost J, *et al.* Four-year injury survey in heptathlon and decathlon athletes. *Sci Sports*. 2012;27:345–50. doi: 10.1016/j.scispo.2012.04.002
39 Edouard P, Depiesse F, Hertert P, *et al.* Injuries and illnesses during the 2011 Paris European Athletics Indoor Championships. *Scand J Med Sci Sports*. 2013;23:213–8. doi: 10.1111/sms.12027
40 Edouard P, Depiesse F, Branco P, *et al.* Analyses of Helsinki 2012 European athletics championships injury and illness surveillance to discuss elite athletes risk factors. *Clinical Journal of Sport Medicine*. 2014;24:409–15. doi: 10.1097/JSM.0000000000000052

41 Edouard P, Alonso J-M, Serra JM, *et al.* Incidences et caractéristiques des blessures lors des Championnats internationaux d'athlétisme 2011 et 2012. *Journal de Traumatologie du Sport*. 2014;31:18–27.
42 Edouard P, Feddermann-Demont N, Alonso JM, *et al.* Sex differences in injury during top-level international athletics championships: Surveillance data from 14 championships between 2007 and 2014. *Br J Sports Med*. 2015;49:472–7. doi: 10.1136/bjsports-2014-094316
43 Edouard P, Morin J-B, Samozino P. No change in maximal lower extremity power output was induced by a decathlon. *Sci Sports*. 2015;30:e73–83. doi: 10.1016/j.scispo.2014.02.005
44 Edouard P, Branco P, Alonso JM. Muscle injury is the principal injury type and hamstring muscle injury is the first injury diagnosis during top-level international athletics championships between 2007 and 2015. *Br J Sports Med*. 2016;50:619–30. doi: 10.1136/bjsports-2015-095559
45 Edouard P, Serra J, Cugy E, *et al.* Prévention des blessures en athlétisme : démarche scientifique par application du modèle de van Mechelen en quatre étapes. *Journal de Traumatologie du Sport*. 2016;33:34–42. doi: 10.1016/j.jts.2015.12.004
46 Edouard P, Junge A, Sorg M, *et al.* Illnesses during 11 international athletics championships between 2009 and 2017: Incidence, characteristics and sex-specific and discipline-specific differences. *Br J Sports Med*. 2019;53:1174–82. doi: 10.1136/bjsports-2018-100131
47 Edouard P, Navarro L, Branco P, *et al.* Injury frequency and characteristics (location, type, cause and severity) differed significantly among athletics ('track and field') disciplines during 14 international championships (2007-2018): Implications for medical service planning. *Br J Sports Med*. 2020;54:159–67. doi: 10.1136/bjsports-2019-100717
48 Edouard P, Navarro L, Pruvost J, *et al.* In-competition injuries and performance success in combined events during major international athletics championships. *J Sci Med Sport*. 2021;24:152–8. doi: 10.1016/j.jsams.2020.07.011
49 Edouard P, Hollander K, Navarro L, *et al.* Lower limb muscle injury location shift from posterior lower leg to hamstring muscles with increasing discipline-related running velocity in international athletics championships. *J Sci Med Sport*. 2021;24:653–9. doi: 10.1016/j.jsams.2021.02.006
50 Edouard P, Pollock N, Guex K, *et al.* Hamstring Muscle Injuries and Hamstring Specific Training in Elite Athletics (Track and Field) Athletes. *Int J Environ Res Public Health*. 2022;19:10992. doi: 10.3390/ijerph191710992
51 Edouard P, Junge A, Alonso JM, *et al.* Having an injury complaint during the four weeks before an international athletics ('track and field') championship more than doubles the risk of sustaining an injury during the respective championship: a cohort study on 1095 athletes during 7 international championships. *J Sci Med Sport*. 2022;25:986–94. doi: 10.1016/j.jsams.2022.10.010
52 Edouard P, Mosser C, Chapon J, *et al.* Understanding the first injury in athletics and its effect on dropout from sport: an online survey on 544 high-level youth and junior athletics (track and field) athletes. *BMJ Open Sport Exerc Med*. 2024;10:e001767.
53 Edouard P, Sorg M, Martin S, *et al.* Athletes who have already experienced an injury are more prone to adhere to an injury risk reduction approach than those who do not: an online survey of 7870 French athletics (track and field) athletes. *BMJ Open Sport Exerc Med*. 2024;10:e001768.

54 Edouard P, Depiesse F. Incidences et caractéristiques des blessures lors des championnats de France élite d'athlétisme de 2014 à 2017. *Journal de Traumatologie du Sport*. 2024;41:73–7. doi: 10.1016/j.jts.2023.10.002
55 Edouard P, Hollander K. Injuries by Events in Combined Events (Decathlon and Heptathlon) During 11 International Outdoor Athletics Championships. *Scand J Med Sci Sports*. 2025;35. doi: 10.1111/sms.70142
56 Enoki S, Nagao M, Ishimatsu S, *et al.* Injuries in Collegiate Track and Field Jumping: A 2-Year Prospective Surveillance Study. *Orthop J Sports Med*. 2021;9:2325967120973397. doi: 10.1177/2325967120973397
57 Fan Y. Decathlon each interaction regression factors analysis based on GRA and FAM. *J Chem Pharm Res*. 2014;6:261–8.
58 Faris AW, Gilley WF, Dean GM, *et al.* Physiological Profiles of world class decathletes in training. *J Sports Med*. 1980;20:285–90.
59 Feddermann-Demont N, Junge A, Edouard P, *et al.* Injuries in 13 international Athletics championships between 2007-2012. *Br J Sports Med*. 2014;48:513–22. doi: 10.1136/bjsports-2013-093087
60 Fisher AG, Adams TI, Yanowitz F 6, *et al.* Noninvasive Evaluation of World Class Athletes Engaged in Different Modes of Training. *Am J Cardiol*. 1989;63:337–41.
61 Ganse B, Degens H, Drey M, *et al.* Impact of age, performance and athletic event on injury rates in master athletics -First results from an ongoing prospective study. *J Musculoskeletal Neuronal Interact*. 2014;14:148–54.
62 García-Roca JA, García-Manso JM, Fuentes-García JP, *et al.* Performance analysis of the events groups as a predictor of high-level decathletes. *Cultura, Ciencia y Deporte*. 2022;17:179–87. doi: 10.12800/ccd.v17i52.1789
63 Gassmann F, Fröhlich M, Emrich E. Structural analysis of women's heptathlon. *Sports*. 2016;4:12. doi: 10.3390/sports4010012
64 Gregor RJ, Edgerton VR, Rozenek R, *et al.* Skeletal Muscle Properties and Performance in Elite Female Track Athletes*. *European Journal of Appliecl Physiology and Qccupational Physiology*. 1981;47:355–64.
65 Hiroshige Y, Watanabe H, Tomiyama S, *et al.* Epidemiological Study of Track-and-Field Meets On-Field Medical Care. *J Sport Rehabil*. 2025;34:102–8. doi: 10.1123/jsr.2023-0316
66 Hollander K, Klöwer M, Richardson A, *et al.* Apparent temperature and heat-related illnesses during international athletic championships: A prospective cohort study. *Scand J Med Sci Sports*. 2021;31:2092–102. doi: 10.1111/sms.14029
67 Horst F, Janssen D, Beckmann H, *et al.* Can Individual Movement Characteristics Across Different Throwing Disciplines Be Identified in High-Performance Decathletes? *Front Psychol*. 2020;11:2262. doi: 10.3389/fpsyg.2020.02262
68 Houtkooper LB, Mullins VA, Going SB, *et al.* Body Composition Profiles of Elite American Heptathletes. *Int J Sport Nutr Exerc Metab*. 2001;11:162–73.
69 Houtkooper L, Abbot JM, Nimmo M. Nutrition for throwers, jumpers, and combined events athletes. *J Sports Sci*. 2007;25:S39–47. doi: 10.1080/02640410701607262
70 Jacobsson J, Timpka T, Kowalski J, *et al.* Prevalence of musculoskeletal injuries in Swedish elite track and field athletes. *American Journal of Sports Medicine*. 2012;40:163–9. doi: 10.1177/0363546511425467

71 Jacobsson J, Timpka T, Kowalski J, *et al.* Injury patterns in Swedish elite athletics: annual incidence, injury types and risk factors. *Br J Sports Med*. 2013;47:986–91. doi: 10.1136/bjsports-2012-091651
72 Jin S, Guo T. Grey forecasting model in the application of world women's pentathlon performance prediction research. *BioTechnology*. 2014;10:4596–605.
73 Karvonen MJ, Niemi N. Factor analysis of performance in track and field events. *Arbeitsphysiologie*. 1953;15:127–33.
74 Kenny IC, Sprevak D, Sharp C, *et al.* Determinants of Success in the Olympic Decathlon: Some Statistical Evidence. *J Quant Anal Sports*. 2005;1:5. doi: 10.2202/1559-0410.1002
75 Kim BY, Vigil D V. A Review of Injury Patterns in Athletes Competing in Combined Competitions: Heptathlon and Decathlon. *Curr Sports Med Rep*. 2016;15:433–6. doi: 10.1249/JSR.0000000000000317
76 Krahenbuhl GS, Wells CL, Brown CH, *et al.* Characteristics of national and world class female pentathletes. *Med Sci Sports*. 1979;11:20–3.
77 Kunz H, Kaufmann DA. Biomechanical analysis of sprinting: decathletes versus champions. *Br J Sports Med*. 1981;15:177–81. doi: 10.1136/bjsm.15.3.177
78 Kunz H, Kaufmann DA. Cinematographical analysis of javelin throwing techniques of decathletes. *Br J Sports Med*. 1983;17:200–4. doi: 10.1136/bjsm.17.3.200
79 Leonhardt TPM, Bristol A, McLaurin N, *et al.* Dietary Intake of Athletes at the World Masters Athletics Championships as Assessed by Single 24 h Recall. *Nutrients*. 2024;16:564. doi: 10.3390/nu16040564
80 Linden M. Factor analytical study of olympic decathlon data. *Research Quarterly of the American Alliance for Health, Physical Education and Recreation*. 1977;48:562–8. doi: 10.1080/10671315.1977.10615462
81 Maïmoun L, Coste O, Puech AM, *et al.* No negative impact of reduced leptin secretion on bone metabolism in male decathletes. *Eur J Appl Physiol*. 2008;102:343–51. doi: 10.1007/s00421-007-0592-7
82 Mayr B, Paar O, Bernett P, *et al.* [Sports injuries and sports damage in decathlon competitors]. *Schweiz Z Sportmed*. 1988;36:39–45.
83 Mullins VA, Houtkooper LB, Howell WH, *et al.* Nutritional status of U.S. elite female heptathletes during training. *Int J Sport Nutr Exerc Metab*. 2001;11:299–314.
84 Nunes JR de A, Ceylan HI, de Almeida-Neto PF, *et al.* The Influence of the ACTN3 R577X Genotype on Performance in Brazilian National-Level Decathlon Athletes: A Pilot Study. *Cells*. 2025;14:782. doi: 10.3390/cells14110782
85 Palamarchuk HJ, Oehrlein CR. Freiberg's Infraction in a Collegiate Heptathlete. *J am Podiatr Med Assoc*. 2000;90:77–80.
86 Panday SB, Pathak P, Moon J, *et al.* Aging induces a step-like change in the motor ability structure of athletes. *Aging*. 2019;11:5276–86.
87 Park J, Zatsiorsky VM. Multivariate Statistical Analysis of Decathlon Performance Results in Olympic Athletes (1988-2008). *International Journal of Sport and Health Sciences*. 2011;5:779–82.
88 Pastre CM, Filho GC, Monteiro HL, *et al.* Lesões desportivas na elite do atletismo brasileiro: estudo a partir de morbidade referida. *Rev Bras Med Esporte*. 2005;11:43–7.

89 Pavlović R. Discipline homogeneity based on the most successful decathlon scoring placement. *Turkish Journal of Kinesiology*. 2017;3:6–11.

90 Pavlović R, Vrcić M, Petrović B. Athletic decathlon: are there differences between the results of decathlon record-holders and their best personal results? *Journal of Physical Education Research*. 2020;7:18–26.

91 Périard JD, Racinais S, Timpka T, *et al.* Strategies and factors associated with preparing for competing in the heat: A cohort study at the 2015 IAAF World Athletics Championships. *Br J Sports Med*. 2017;51:264–71. doi: 10.1136/bjsports-2016-096579

92 Gerry Purdy J, White SR. Scoring a decathlon with a portable minicomputer. *Research Quarterly of the American Alliance for Health, Physical Education and Recreation*. 1976;47:860–3. doi: 10.1080/10671315.1976.10616755

93 Remmel L, Ben-Zaken S, Meckel Y, *et al.* The Genetic Basis of Decathlon Performance: An Exploratory Study. *The Journal of Strength and Conditioning Research*. 2023;37:1660–6.

94 Reuter S, Forkel P, Imhoff AB, *et al.* Postural control in elite decathlon athletes: Are various modes of dynamic assessment needed? *Journal of Sports Medicine and Physical Fitness*. 2017;57:936–41. doi: 10.23736/S0022-4707.16.06416-1

95 Ruff CB. Body Mass Prediction From Skeletal Frame Size in Elite Athletes. *Am J Phys Anthropol*. 2000;113:507–17.

96 Samia BAA, Youssef GA. Changes in urinary 8-hydroxydeoxyguanosine levels during heptathlon race in professional female athletes. *J Hum Kinet*. 2014;41:107–11. doi: 10.2478/hukin-2014-0038

97 Schomaker M, Heumann C. Model averaging in factor analysis: An analysis of olympic decathlon data. *J Quant Anal Sports*. 2011;7:4. doi: 10.2202/1559-0410.1249

98 Shirreffs SM, Casa DJ, Carter R. Fluid needs for training and competition in athletics. *J Sports Sci*. 2007;25:S83–91. doi: 10.1080/02640410701607353

99 Sygo J, Killer SC, Glass AK, *et al.* Fueling for the field: Nutrition for jumps, throws, and combined events. *Int J Sport Nutr Exerc Metab*. 2019;29:95–105. doi: 10.1123/ijsnem.2018-0272

100 Tscholl P, Alonso J-M, Dollé G, *et al.* The use of drugs and nutritional supplements in top-level track and field athletes. *American Journal of Sports Medicine*. 2010;38:133–40. doi: 10.1177/0363546509344071

101 Van Damme R, Wilson RS, Vanhooydonck B, *et al.* Performance constraints in decathletes. *Nature*. 2002;415:755–6.

102 Walker JA, Caddigan SP. Performance trade-offs and individual quality in decathletes. *Journal of Experimental Biology*. 2015;218:3647–57. doi: 10.1242/jeb.123380

103 Wang Z, Lu G. The Czech phenomenon of Men's Decathlon developpment. *International Journal of Sports Science and Engineering*. 2007;1:209–14.

104 Ward P, Sprevak D, Boreham C. It takes ten events to make the Olympic decathlon. *Int J Math Educ Sci Technol*. 2002;33:926–33.

105 Wentz S, Engelhardt M, Wentz S. Verletzungsanalyse und Leistungsparameter bei Jugendzehnakaempfern. *Dtsch Z Sportmed*. 1997;48:389–94.

106 Wimmer V, Fenske N, Pyrka P, *et al.* Exploring Competition Performance in Decathlon Using Semi-Parametric Latent Variable Models. *J Quant Anal Sports*. 2011;7:6.
107 Withers RT, Craig NP, Bourdon PC, *et al.* Relative body fat and anthropometric prediction of body density of male athletes*. *Eur J Appl Physiol Occup Physiol*. 1987;56:191–200.
108 Woolf A, Ansley L, Bidgood P. Grouping of Decathlon Disciplines. *J Quant Anal Sports*. 2007;3:5. doi: 10.2202/1559-0410.1057
109 Yoshitake R, Ogata H, Omi N. Blood Glucose Levels during Decathlon Competition: An Observational Study in Timing of Intake and Competing Time. *Metabolites*. 2024;14:47. doi: 10.3390/metabo14010047
110 Zhang Y, Liu Y. Chinese and foreign women heptathlon top athlete competitive ability features comparative research. *J Chem Pharm Res*. 2014;6:984–90.
111 Zhang P, Lu J. Chinese and foreign men's decathlon performance comparison and structural factor correlation test based on SPSS regression model. *BioTechnology Indian Journal*. 2014;10:441–9.

Online supplemental Figure 1. Number of published articles according to the year of publication for the 111 included articles.

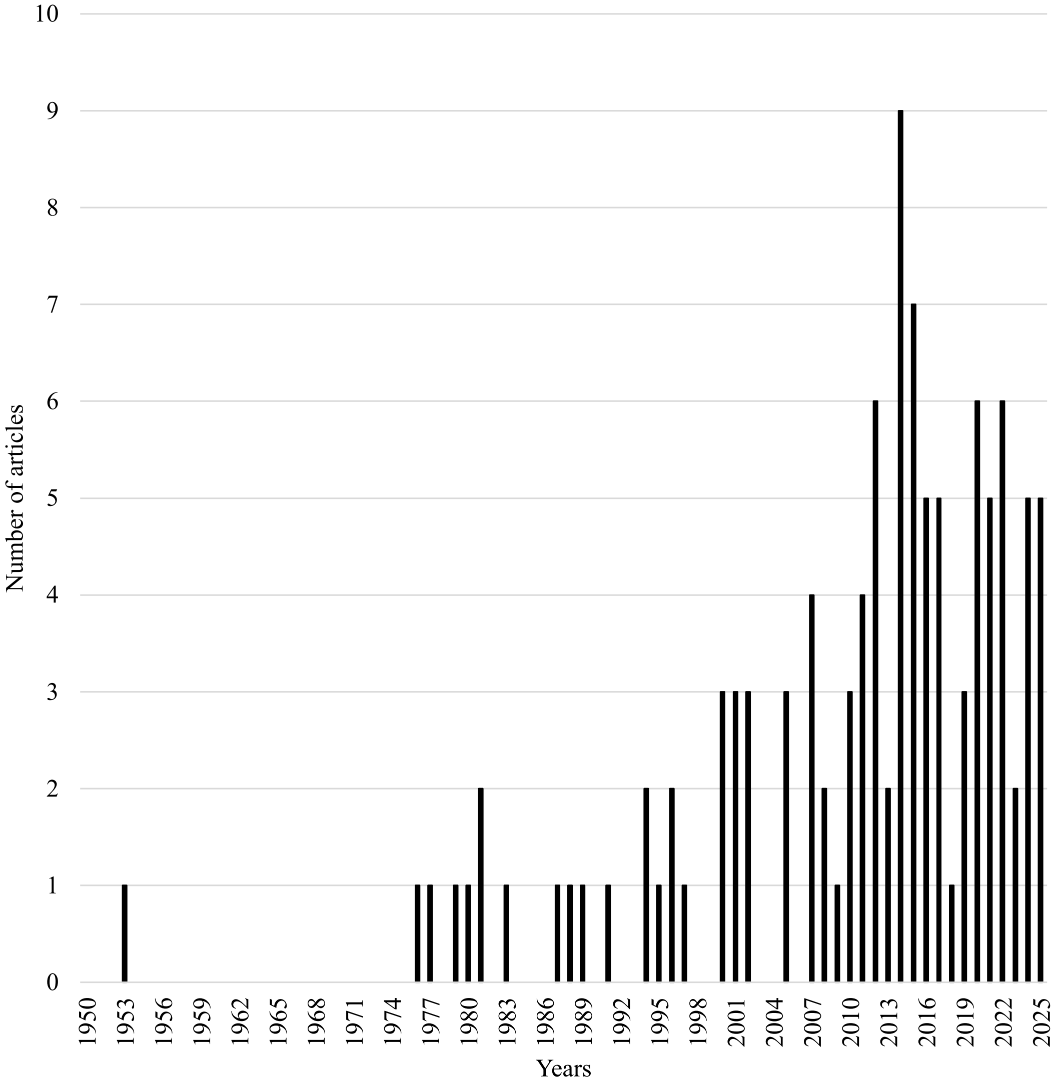

Online supplemental Figure 2. Number of published articles according to continents and countries for the 111 included articles.

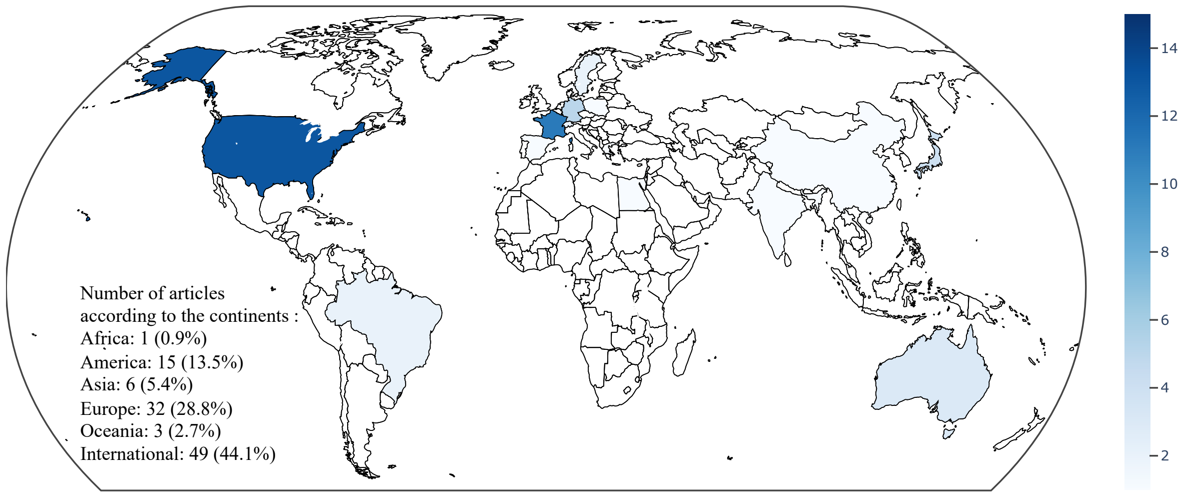

Supplement: Supplementary file 1 — Data S1: Supporting Information [file SMS-36-e70190-s001.pdf]
